# Myocarditis and Inflammatory Cardiomyopathy in Dilated Heart Failure

**DOI:** 10.3390/v17040484

**Published:** 2025-03-27

**Authors:** Francesco Nappi

**Affiliations:** Department of Cardiac Surgery, Centre Cardiologique du Nord, 93200 Saint-Denis, France; francesconappi2@gmail.com or f.nappi@ccn.fr; Tel.: +33-149334104; Fax: +33-149334119

**Keywords:** myocarditis, inflammatory cardiomyopathy, dilated cardiomyopathy, heart failure

## Abstract

Inflammatory cardiomyopathy is a condition that is characterised by the presence of inflammatory cells in the myocardium, which can lead to a significant deterioration in cardiac function. The etiology of this condition involves multiple factors, both infectious and non-infectious causes. While it is primarily associated with viral infections, other potential causes include bacterial, protozoal, or fungal infections, as well as a wide variety of toxic substances and drugs, and systemic immune-mediated pathological conditions. In spite of comprehensive investigation, the presence of inflammatory cardiomyopathy accompanied by left ventricular dysfunction, heart failure or arrhythmia is indicative of an unfavourable outcome. The reasons for the occurrence of either favourable outcomes, characterised by the absence of residual myocardial injury, or unfavourable outcomes, marked by the development of dilated cardiomyopathy, in patients afflicted by the condition remain to be elucidated. The relative contributions of pathogenic agents, genomic profiles of the host, and environmental factors in disease progression and resolution remain subjects of ongoing discourse. This includes the determination of which viruses function as active inducers and which merely play a bystander role. It remains unknown which changes in the host immune profile are critical in determining the outcome of myocarditis caused by various viruses, including coxsackievirus B3 (CVB3), adenoviruses, parvoviruses B19 and SARS-CoV-2. The objective of this review is unambiguous: to provide a concise summary and comprehensive assessment of the extant evidence on the pathogenesis, diagnosis and treatment of myocarditis and inflammatory cardiomyopathy. Its focus is exclusively on virus-induced and virus-associated myocarditis. In addition, the extant lacunae of knowledge in this field are identified and the extant experimental models are evaluated, with the aim of proposing future directions for the research domain. This includes differential gene expression that regulates iron and lipid and metabolic remodelling. Furthermore, the current state of knowledge regarding the cardiovascular implications of severe acute respiratory syndrome coronavirus 2 (SARS-CoV-2) infection is also discussed, along with the open questions that remain to be addressed.

## 1. Introduction

Inflammatory cardiomyopathy is delineated as myocarditis in conjunction with heart impairment and ventricular remodelling [1,2,3,4,5]. Although there has been substantial investigation and enhanced diagnostic methods and comprehension of the underlying pathophysiology of inflammatory cardiomyopathy, this condition remains associated with a poor prognosis when accompanied by left ventricular (LV) dysfunction, heart failure (HF) or arrhythmia [6,7]. In addition to these conditions, the rare instance of full-blown myocarditis, characterised by an abrupt and severe inflammatory response within the heart, has been identified as a primary causative factor of cardiogenic shock in young adults [8,9,10,11,12,13]. The study, which compared fulminant and acute non-fulminant myocarditis in patients with left ventricular systolic dysfunction, established the optimal strategy for the recognition and initial management of myocarditis. It is imperative to note that an accurate diagnosis and precise therapeutic measures are paramount in reducing mortality and the necessity for heart transplantation in these patients [8,9,10,11,12,13]. A plethora of enquiries persist and pertain to the pathogenesis of inflammatory cardiomyopathy, particularly with regard to its progression and prognosis. The role of viral infection, the host genetic background, and environmental factors in disease progression and prognosis remain subjects of significant research interest. These lacunae in our understanding underscore the necessity for the development of sophisticated experimental systems that can more accurately model the human immune system. Moreover, there is a compelling need to enhance the characterisation and classification of patients, employing methodologies such as phenotyping and phenomics. These approaches entail conducting a comprehensive evaluation of immune status, viral presence, and other biomarkers. In detail, a comprehensive MEDLINE, Embase and Cochrane Library search was performed using the search terms “myocarditis” or “viral myocarditis” together with “epidemiology”, “pathogenesis”, “manifestations”, “imaging”, “treatment”, “surgery” or “device”. Publications from the past decade were mostly selected, but widely referenced and highly regarded older publications were also included. Additionally, the reference lists of the selected articles were reviewed thoroughly to identify further relevant sources. Recommended review articles are provided for readers to access additional context and background references.

In this critical appraisal, a thorough examination of the extant literature is undertaken to identify the prevailing gaps in our understanding of the etiology, diagnosis, treatment and prognosis of myocarditis and inflammatory cardiomyopathy. Furthermore, a comprehensive appraisal of the available animal and cell models of these conditions is presented, along with a series of recommendations for potential future research initiatives within this discipline. This study explores the function of viruses as both active inducers and potential bystanders in the pathogenesis of myocarditis and evolutionary myocardial inflammation onto cardiomyopathy. The study goes on to evaluate the significance of histology and immunohistology, as well as the use of molecular biology techniques for analysing endomyocardial biopsy (EMB) samples. In addition to this, it also analyses state-of-the-art imaging procedures and the role of inflammatory and immune cell markers. Moreover, the study proceeded to analyse the role of immune cell ratios, microRNAs and antibodies in the diagnosis, direction of therapeutic interventions and oversight of subjects diagnosed with myocarditis and concurrent inflammatory myocardial disease. In this study, patient-specific therapeutic options are delineated, with said options grounded in an accurate diagnosis. Current as well as novel strategies are thus covered. This review is intended to aid medical practitioners and scientists in the implementation of optimal methodologies for diagnosis and treatment, with the objective of resolving patient-specific issues within a clinical context.

## 2. Viruses’ Position in Pathogenesis

Myocarditis is defined as an inflammatory heart condition caused primarily by viral infections [14,15], with other potential triggers including bacteria (e.g., Borrelia spp.), protozoa (e.g., Trypanosoma cruzi), and fungi. Additional risk factors for myocarditis include a broad range of toxic chemicals and pharmaceutical agents (e.g., immune checkpoint inhibitors) [16] and systemic immune-mediated diseases [17]. A salient point is the significant variability in the aetiogenesis, induction and course of myocarditis in relation to diverse infectious agents.

### 2.1. Viral Agents

**Figure 1:**
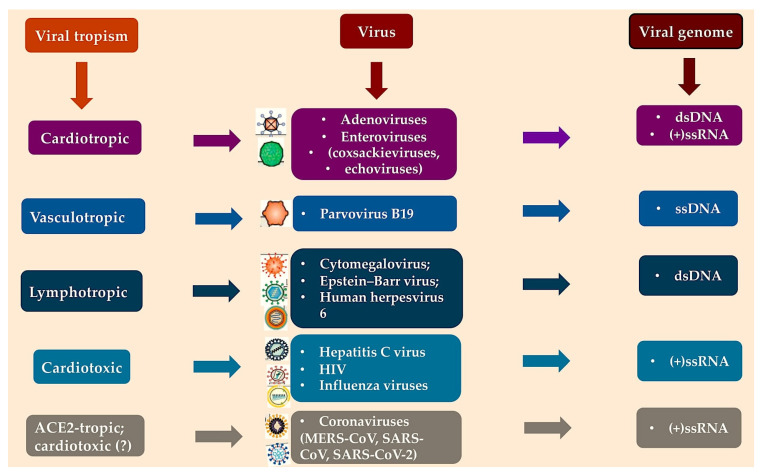
The following illustration is of viruses associated with myocarditis and inflammatory cardiomyopathy: Viral tropism, viral agent and viral genome are represented. The manner in which viruses attach to and infect different tissues is specific. For example, they have the capacity to attach to and infect heart tissue (cardiotropic), blood vessels (vasculotropic), lymph tissue (lymphotropic), heart tissue (cardiotoxic) and the ACE2 receptor (ACE2 receptor tropism). The viral genome is identified in the tissue [4,10,21,22,23,24,25,26,27,28,29,30,31,32,33,34,35].

There are three main categories of virus that cause inflammatory cardiomyopathy. The primary category includes cardiotropic viruses that can be eliminated from the heart. Examples include adenoviruses and enteroviruses, such as coxsackie A and B viruses and echoviruses. Second are the vasculotropic viruses that are likely to persist lifelong, such as parvovirus B19 from the erythrovirus family. Third are the lymphotropic viruses belonging to the Herpesviridae family, which include human herpesvirus 6 (HHV6), Epstein–Barr virus and human cytomegalovirus. Viral agents can also trigger myocarditis through indirect immune system activation [18,19]. It is clear that the following viruses are listed: human immunodeficiency virus (HIV), hepatitis C virus (HCV), influenza A virus and influenza B virus; and viruses of the Coronaviridae genre, which include Middle Eastern respiratory syndrome-associated Coronavirus (MERS-CoV). These are the viruses that cause severe acute respiratory syndrome (SARS), SARS-CoV and SARS-CoV-2, all of which can exert direct injury to the heart via angiotensin-converting enzyme 2 (ACE2) tropism. Coronaviruses are known to cause myocarditis indirectly via cytokine-mediated cardiotoxicity or the induction of an autoimmune response against cardiac components (Figure 1) as is the case with influenza A and B viruses [20], Figure S1A,B in Supplementary Materials.

The exact pathomechanisms underpinning SARS-CoV-2-associated heart disease remain poorly understood, and await detailed examination of EMB and postmortem specimens from infected individuals.

It is imperative to delineate between virus-induced inflammatory cardiomyopathy and virus-associated inflammatory cardiomyopathy (in the event of viral latency). This dichotomy is contingent on the causal relationship between the virus and the pathogenesis of inflammatory cardiomyopathy. It is also vital to set up a full classification system. This system must clearly define the differences in viruses that directly (cardiotropic and vasculotropic viruses) or indirectly (lymphotropic viruses) infiltrate the heart (Figure S1A Supplementary Materials). It must also distinguish between viruses that do not necessitate directly infecting cardiac cells. Instead, these viruses can indirectly trigger cardiac damage and negative inotropy through the activation of a cytokine response or a cellular immune reaction involving molecular mimicking.

It is important to note that there are some discrepancies between the International Guidelines that require further clarification. The ESC Guidelines mandate viral diagnostics [10], encompassing the analysis of the viral genome in EMB samples via quantitative PCR, to ascertain the underlying causative factors of inflammatory cardiomyopathy. In contrast to this, the AHA does not recommend conducting standard viral genome testing for the diagnosis of inflammatory cardiomyopathy [21], although this technique is mentioned in a 2020 scientific statement as a potentially valuable approach in instances where a definitive diagnosis is unclear [10]. It is evident that additional studies are necessary to ascertain and substantiate the function of viral genome identification in the heart for the purposes of diagnosis and management of inflammation-induced cardiomyopathy (Figure S1B Supplementary Materials).

In recent decades, a notable increase in the prevalence of B19V and HHV6 has been observed in endomyocardial biopsy (EMB) samples from patients diagnosed with myocarditis when compared with other enteroviruses and adenoviruses. Approximately 30% of these cases have been found to have co-infections, with multiple viruses being detected in the same patient sample [18,22]. Furthermore, a significant increase in the prevalence of acute enterovirus myocarditis has been observed in the paediatric population over the course of the last five years [23,24,25]. The temporal variation in the detection frequency of viruses associated with inflammatory cardiomyopathy is a subject of ongoing research. The evaluation of a broader repertoire of viruses has had a considerable influence on these variations, Figure S2 Supplementary Materials [26].

A considerable number of viral ailments are distinguished by seasonal distribution. For instance, the prevalence of influenza viruses is highest during the colder periods of the year, whereas the prevalence of enteroviruses is observed to be highest during the more temperate summer and autumn seasons [27]. However, it is acknowledged that variations in climatic conditions across different regions can influence the seasonal patterns of viral infections [27]. Enteroviral myocarditis is a prevalent condition among male adolescents and adults [28,29]. Furthermore, male sex has been identified as a significant risk factor for mortality in patients with coronavirus disease 2019 (COVID-19) [30,31], a condition caused by SARS-CoV-2 infection. This observation suggests that the outcome of cardiovascular disease associated with viral infection may be influenced by differences in immune responses between males and females [32,33,34,35].

- Coronaviridae

Coronaviruses, which are classified within the Coronaviridae genus, are categorised into four distinct taxonomic categories: Alphacoronavirus, Betacoronavirus, Gammacoronavirus and Deltacoronavirus. Among these, Alphacoronavirus and Betacoronavirus have been identified as the causative agents of human infections [36]. As demonstrated in the current literature, various strains of the Coronaviridae family are continually present within the human population, predominantly manifesting as benign respiratory ailments [37]. In contrast, Middle East Respiratory Syndrome (MERS)-Coronavirus (MERS-CoV), Severe Acute Respiratory Syndrome (SARS)-Coronavirus (SARS-CoV), and Middle East Respiratory Syndrome (MERS)-Coronavirus-2 (SARS-CoV-2) have the capacity to propagate from animals to humankind, resulting in severe respiratory maladies [38]. It has been demonstrated that advanced age (defined as >60 years), male sex, and the presence of comorbidities (including hypertension and obesity) are recognised as the primary risk elements for fatal outcomes in patients with confirmed cases of the novel coronavirus [39,40,41,42,43]. Cardiac injury, defined by elevated plasma troponin levels, increased plasma d-dimer levels or IL-6 levels, and acute respiratory distress syndrome, have been identified as independent factors associated with mortality in patients with confirmed or suspected cases of the infection [30,31,41,42,43] The suggested mechanisms of myocardial injury in patients with COVID-19 include the following. Firstly, myocardial damage by a cytokine storm initiated by an impaired reaction of T helper 1 cells (TH1 cells) and T helper 2 cells (TH2 cells) [44,45,46]. Secondly, respiratory dysfunction and hypoxaemia resulting from SARS-CoV-2 infection [43,47]. Furthermore, the potential for myocardial damage arising from reduced function of the ACE2-angiotensin (1–7) pathway, a system with cardioprotective properties within the renin-angiotensin system, is a plausible consideration [48,49]. Levels of both the enzyme angiotensin-converting enzyme 2 and the hormone angiotensin (1–7) have been demonstrated to decrease in autopsy samples from patients who tested positive for SARS-CoV and SARS-CoV-2. Moreover, ACE2 has been identified as the key doorway for the entrance of various coronaviruses, including SARS-CoV and SARS-CoV-2, into the cells of their hosts [50,51]. The binding of the viral spike protein to ACE2, and the subsequent priming of the spike protein by the host cell serine proteases TMPRSS2, cathepsin B and cathepsin L, are essential for the viral entry mechanism [51,52]. It is noteworthy that TMPRSS2, a key player in this intricate interplay, is expressed in lung cells that are known to express high levels of ACE2, underscoring its critical role in facilitating viral entry [51]. In a recent study, Nicin and colleagues demonstrated that cardiac cells, encompassing cardiomyocytes, pericytes, fibroblasts, endothelial cells, and leukocytes, isolated from patients afflicted with HF with decreased ejection fraction or aortic stenosis, exhibited the presence of ACE2 [53]. Consistent with these findings, Tschöpe et al. [54] recently reported that certain review contributors observed, in a single EMB sample from a patient with DCM, that ACE2 is predominantly expressed in cardiomyocytes, pericytes and fibroblasts, although these cardiac cells did not express TMPRSS2. However, the authors noted that these observations were unpublished. In addition, the presence of SARS-CoV-2 has been identified in cardiac tissue, suggesting the potential for the virus to infiltrate the heart during periods of transient viraemia or through the process of infected macrophages infiltrating the myocardium. In the context of the investigation of the myocardial tissue of patients with confirmed cases of the disease, the presence of viral particles within the endothelial cells of blood vessels and an accumulation of inflammatory cells in the myocardium has been reported. This is indicative of the presence of endotheliitis and the subsequent death of inflammatory cells. Notwithstanding, the classic form of acute lymphocytic myocarditis or lymphocytic inflammatory cardiomyopathy has hitherto not been detected in patients with COVID-19 [20]. In order to establish an appropriate classification of the associated heart disease, further investigation is required into the mechanisms by which SARS-CoV-2 infection causes myocardial damage.

- Adenoviruses and enteroviruses

Enteroviruses, predominantly coxsackie B viruses and a number of adenoviruses, have been identified as causative agents of acute myocarditis and the subsequent development of inflammation within the heart muscle [27]. These viruses infect the heart muscle cells through interaction with a specific transmembrane receptor, known as the coxsackievirus and adenovirus receptor (CAR), which facilitates viral entry [55]. This process can result in direct myocardial injury, including disruption of the cell’s cytoskeleton [56], and may also initiate an excessive immune response, even after the initial viral infection has been resolved. These viruses are classified as members of the family of cytolytic agents, which are characterised by their ability to trigger myocarditis through a process involving viral replication within the infected cell, leading to the subsequent cell lysis and secretion of the virus. It has been demonstrated that the prolonged presence of adenoviruses and enteroviruses within the myocardium can result in left ventricular dysfunction, suboptimal clinical outcomes, and an elevated mortality rate among patients afflicted with this condition [18,57]. Conversely, approximately 50% of patients with heart inflammation caused by enterovirus or adenovirus show full resolution without any lasting damage, leading to a successful recovery [58]. Research has demonstrated that patients carrying the CCR5Δ32 deletion (which is associated with a lack of CC-chemokine receptor 5, or CCR5) either in heterozygosity or homozygosity, have been observed to display an increased capacity to resolve enterovirus infections when compared to patients carrying the wild-type CCR5 [57]. This finding underscores the pivotal role that an individual’s genetic makeup plays in determining the progression and outcome of a disease. In addition, mutant variants of coxsackievirus B3 (CVB3) with 5′ terminal deletions in their genomic RNA have been identified in a patient suffering from idiopathic dilated cardiomyopathy (DCM). This mutation has been proposed as a potential mechanism for viral persistence within the heart [59]. A subsequent study revealed that prolonged existence of group B enteroviruses is characterised by a 5′ terminal deletion in their genomic RNA, and that these viruses can compromise cardiomyocyte function through the proteolytic activity of viral proteinase 2A in patients with unexplained DCM [60]. While this study offers a potential explanation for viral perpetuation, it also suggests that conventional PCR might not be sufficiently sensitive to detect these mutant viruses [61]. It is evident that this virulent pathogen has the ability to enter the endothelial cells and trigger the release of pro-inflammatory cytokines, a phenomenon that is facilitated by the toxic, non-structural viral protein known as NS1 [61]. Consequently, B19V infection of endothelial cells has the capacity to induce cardiomyoctye apoptosis, a phenomenon that has been demonstrated in vitro in B19V-infected endothelial cells co-cultured with cardiomyocytes [55,62,63,64].

- Parvovirus B19

B19V infection presents a broad clinical spectrum. While usually mild and self-limiting, B19V infection can also induce severe septic and haematological complications. B19V has been shown to cause acute cardiac infection in cases of severe viraemia [62], and can also remain in the heart, with recurrent episodes of viral replication [14]. It is well-documented that the persistence of severe cardiac inflammation, especially in the setting of acute B19V infection, can lead to cardiomyocyte necrotisation [65]. The repercussions of B19V latency within the cardiac tissue on clinical outcomes continue to be a subject of discourse [66,67]. The etiopathological role of B19V infection in the genesis of myocarditis remains ambiguous. This is predicated on the frequent detection of B19V in myocardial autopsy samples from subjects without myocarditis or dilated cardiomyopathy [68,69]. It can therefore be hypothesised that the detection of B19V DNA in cardiac tissue may signify that B19V is a non-specific concomitant of myocarditis, rather than the primary causative agent of the illness [70]. The prevailing view is that the occurrence of elevated copy numbers of B19V DNA (in excess of 500 viral DNA copies per microgram of cardiac DNA) in cardiac tissue is associated with myocarditis [71]. The evidence base for this association is supported by findings that include, but are not limited to, the presence of a high B19V DNA copy number in the heart [72,73], the presence of active replication of B19V with detectable viral RNA [74,75], and the concomitant presence of lymphotropic viruses (e.g., HHV6) [72]. However, further research is necessary to confirm these findings [74,75]. The preponderance of EMB specimens from patients diagnosed with acute myocarditis or inflammatory cardiomyopathy exhibiting diminished levels of B19V DNA prompts further inquiry into the potential aetiopathogenic implications of persistent B19V infection as a trigger for the onset of chronic inflammation-mediated cardiac disease.

- Herpesviridae

It is a well-established fact that viruses belonging to the Herpesviridae family, including Epstein–Barr virus, HHV6, and CMV, have the ability to persist indefinitely within the human body. Indeed, Epstein–Barr virus has been documented to trigger a severe, chronic active infection of CD8+ T cells in the myocardium of a patient with persistent perimyocarditis [76]. It is important to note that the most prevalent cardiac herpesvirus, HHV6, which similarly affects T cells, is classified into the subgroups HHV6A and HHV6B. A salient feature of the HHV6 genome is its ability to incorporate into the DNA of somatic cells or germ line cells [77]. However, further research is needed to understand if the recurrence of integrated HHV6 copies can trigger myocarditis.

- HIV, hepatitis C virus and influenza A and B viruses.

The present study hypothesises that myocarditis arising from HIV [78], HCV [79] or influenza virus [80] infections may be attributable to immune-mediated effects. The findings indicate a correlation between the persistence of HCV infection and the subsequent development of DCM, and the genetic background of the patients. Specifically, the analysis reveals that HLA-DPB1*0901 and HLA-DRB1*1201 alleles demonstrate higher prevalence in these patients [79].

Gaps to fill and future directions to explore pertaining to the virus responsible for myocarditis are documented in Supplementary Materials Table S1.

### 2.2. Immune Cells’ Function

The comprehension of immune cell function in the context of inflammatory cardiomyopathy is a subject that is the focus of ongoing research. The significance of immune cells in the development of viral myocarditis and viral inflammatory cardiomyopathy has been substantiated in experimental murine models [81].

The aetiopathogenesis of viral inflammatory cardiomyopathy (VICM) may be conceptualised in terms of three phases. The initial phase is an acute phase of viral entry into the cell and the subsequent activation of innate immune response, which may endure for a period of 1–7 days. This is succeeded by a subacute phase characterised by the activation of adaptive immune response, which can persist for a duration of 1–4 weeks. The final phase of VICM is a chronic phase that can extend from months to years. In this phase, delayed or ineffective viral clearance, in conjunction with chronic inflammation and cardiac remodelling, has been demonstrated to be a contributing factor to DCM [82].

It has been established that the host’s innate immune system is initiated in response to the infection. The innate immune response involves activation of innate immune cells, as well as cardiac cells including cardiomyocytes. This activation is achieved by the recognition of pattern recognition receptors, such as Toll-like receptors (TLRs) [83] and nucleotide-binding oligomerisation domain-like receptors (NODLs), [84,85] which recognise pattern motifs found on pathogens. These receptors are able to recognise specific molecular patterns of pathogens (termed pathogen-associated molecular patterns (PAMPs)) and patterns released from damaged cells (termed damage-associated molecular patterns (DAMPs)), such as released ATP, S100A8 and S100A9 [86]. The nature of the signalling receptor and the subsequent downstream response can vary according to the specific pathogen or DAMP involved. Following activation of innate immune cells and cardiac tissue, a range of pro-inflammatory molecules are released, including cytokines, chemokines, interferons and alarmins. These molecules contribute to the further activation and homing of innate immune cells to the heart, including mast cells, neutrophils, dendritic cells, monocytes and macrophages [87]. In human and experimental myocarditis, monocytes and macrophages are the principal cell sub-types responsible for inflammation [87]. Whilst the initiation of the innate immune response in the heart is advantageous to the host due to the resulting virucidal properties, over-activation or sustained activation of the innate immune system can result in an exacerbated and/or chronic inflammatory process, leading to myocardial injury and remodelling, and ultimately resulting in heart function impairment [88].

Damage to the heart can trigger a number of biological responses, including the release of danger signals such as alarmins and IL-1β. These signals initiate a process of emergency haematopoiesis in the bone marrow, leading to the formation of monocytes. These monocytes and myeloid progenitor cells then migrate to the spleen, where further monocyte production takes place [89,90]. As a consequence, the pool of pro-inflammatory monocytes in the spleen undergoes replenishment and can be mobilised to the damaged heart. The process by which immune cells, principally monocytes, home from the spleen to the heart, the designated cardiosplenic axis, has been the subject of detailed investigation in the setting of ischaemic heart muscle dysfunction [91,92]. The significance of the cardiosplenic pathway in the context of inflammation-induced cardiomyopathy, along with its role as a target for modulating the trafficking of immune cells to the heart, has been elucidated through investigations in murine models of CVB3-induced myocarditis. In this mouse model, the attenuation of the CCR2–CCL2 axis, a key player in the recruitment of pro-inflammatory Ly6C^high^ monocytes to the heart, has been shown to result in a significant alleviation of myocarditis [93,94,95,96]. Conversely, the disruption of the CX3C chemokine receptor 1 (CX3CR1)-CX3C chemokine ligand 1 (CX3CL1) axis has been shown to exacerbate CVB3-induced myocarditis [97]. This pathway plays a crucial role in the attraction of monocytes that are implicated in the inflammatory response. Of particular interest is the finding that the spleen serves as a target organ for CVB3 in mice, with mouse splenic B cells, CD4+ T helper (TH) cells and macrophages and monocytes expressing αMβ2 integrin (also known as CD11c) as the target cells for CVB3 [98]. In addition, human monocytes have been identified as target cells for CVB3 [99]. It can thus be deduced that the migration of CVB3-infected immune cells into the heart can lead to an exacerbation of cardiac viral load and chronic systemic inflammatory response [94,96,100]. One potential therapeutic approach to mitigating this process involves the modulation of the cardiosplenic axis. This can be achieved through the administration of therapies that reduce monocytopoiesis, such as IL-1β inhibitors [90], or by the transfer of regulatory T cells (Treg cells) [96] and mesenchymal stromal cells [85,94,101]. These cells have been shown to block the recruitment of pro-inflammatory monocytes into the heart. As demonstrated in Table 1, a range of immunocells is reported, along with their substantial role in relation to the viruses under discussion [17,82,93,94,95,96,97,98,99,100,101,102,103,104,105,106,107,108,109,110,111,112,113,114,115,116,117,118,119,120,121,122,123,124,125,126,127,128,129,130,131,132,133,134].

There is a lack of research on the role of B cells in the progression of myocarditis to dilated cardiomyopathy. However, the existing literature suggests the presence of infected, activated B cells in the heart tissue of CVB3-infected immunocompetent mice and in severe, coupled immunodeficient mice following the administration of splenocytes from CVB3-infected, syngeneic sources. This observation supports the hypothesis that B cell trafficking may contribute to the maintenance of chronic inflammatory heart disease [100]. B cells are a critical link between the innate and adaptive immune systems, equipped with antigen-specific B cell receptors and TLRs. The connection between TLR signals and B cell activation and tolerance, along with various pathological conditions such as viral myocarditis and septic cardiomyopathy, has been thoroughly examined in the research community. The significant body of evidence concerning the role of B cells in inflammatory cardiomyopathy is largely attributable to the characterization of autoantibodies associated with DCM [127]. Autoantibodies, including those directed against β1-adrenergic receptor, mitochondrial constituents, cardiac myosin heavy chain isoforms, cardiac troponin, Na+/K+-ATPase, and other cardiorelated proteins, have been implicated in the pathogenesis of DCM, potentially leading to heart failure. Moreover, the exhaustive inquiry of subjects diagnosed with subacute or chronic inflammatory myocarditis has indicated that CD20+ B cells—the role of which has been established as the inducer of myocardial impairment in murine models through T cell activation [128] and monocyte mobilization [129]—may offer a potential explanation for the pathophysiology of inflammatory cardiomyopathy [130]. As outlined in Supplementary Materials Table S2, there are gaps to fill and future directions to explore regarding the function of immune cells.

### 2.3. Myocarditis Autoimmunity Profile

The significance of autoimmunity in the aetiology of inflammatory cardiomyopathy has been comprehensively documented in the extant scientific literature. The diagnostic criteria for organ-specific autoimmune diseases, as delineated by Rose–Wittek, are fulfilled by inflammatory cardiomyopathy, which is typified by the presence of immune cell infiltrates and abnormal expression of HLA class II and/or adhesion molecules, in the absence of viral genomes in endomyocardial biopsy (EMB) samples from both index cases and family relatives. As demonstrated in the scientific literature, the presence of autoimmunity is a contributing factor to the development of inflammatory cardiomyopathy [135]. The diagnostic criteria for organ-specific autoimmune diseases, as outlined by Rose–Wittek, are met by inflammatory cardiomyopathy, characterised by the presence of immune cell infiltrates and abnormal expression of HLA class II and/or adhesion molecules, in the absence of viral genomes in endomyocardial biopsy (EMB) samples from both index cases and family relatives. Circulating cardiac autoantibodies in subjects with inflammatory cardiomyopathy and their family members [4,136,137,138]; the availability of animal studies of experimental models of inflammatory cardiomyopathy with or without a DCM phenotype. The availability of animal studies of experimental models of inflammatory cardiomyopathy with or without a DCM phenotype after immunisation with specific autoantigen(s) [4,139,140,141] is also a subject of study. In addition, the reaction to immunosuppression or immunomodulation in subjects with virus-negative-induced inflammatory cardiomyopathy [4,142,143,144] is also a subject of study.

- Autoantibodies specific to the heart

The presence of heart-specific autoantibodies has been documented in up to 60% of patients diagnosed with inflammatory cardiomyopathy, as well as in their family members [4,145,146]. These autoantibodies have been observed to bind to a variety of cardiac autoantigens, with a particular focus on cardiac α-myosin heavy chain (also referred to as myosin 6) and β-myosin heavy chain (also referred to as myosin 7) isoforms [147]. It appears that a number of the autoantibodies in question possess a direct pathogenic and/or prognostic role [148,149]. The administration of autoantigens to animals, which have been identified in patients with inflammatory cardiomyopathy, such as the β1-adrenergic receptor, the muscarinic acetylcholine receptor M2, cardiac myosin heavy chain isoforms and cardiac troponin, [139,140,141,150,151,152] has been shown to result in cardiac abnormalities that mirror the human manifestation of the disease. The process of passive transmission of antibodies derived from rats that have been immunised with cardiac myosin has been observed to result in the deposition of antibodies within the myocardium, leading to the subsequent apoptosis of myocytes and the subsequent development of cardiomyopathy in recipient animals [141]. As demonstrated in experimental models using animals, the presence of both immune-mediated and cell-mediated forms of inflammatory cardiomyopathy has been shown to be evident. However, the prevalence of both forms in patients remains to be determined. This is because the majority of studies conducted on patients with inflammatory cardiomyopathy have focused on investigating humoral immune processes as opposed to cellular pathways.

- How genes interact with the environment

Autoimmune inflammatory cardiomyopathy (AIFM) has been observed in the setting of systemic immune-mediated diseases [17] or may be iatrogenic, i.e., induced by immune suppression therapy [16]. Further research is required to determine whether AIFM has a hereditary component, as indicated by a genetic association study in patients with dilated cardiomyopathy [153]. This research established a risk locus for idiopathic DCM, which has been shown to encode HLA class I and HLA class II proteins. This suggests the hypothesis that genetically driven autoimmune inflammatory processes may have a role in the pathogenesis of idiopathic DCM [153]. Following the MOGE(S) classification [154,155], it is proposed that autoimmune inflammatory cardiomyopathy represents a prevalent terminal stage resulting from the interplay of multiple aetiological factors within a multifactorial sequence encompassing gene–environment interactive processes [156]. For instance, patients diagnosed with myocarditis exhibit demonstrable immune reactivity to both myosin 6 antigens and myosin peptide mimics derived from the gut microbiota, specifically Bacteroides species [157]. These findings align with the gene–environment interaction model and suggest that targeting the microbiome of individuals with a genetic predisposition for myocarditis may lead to a reduction in disease acuity and, consequently, may contribute to the mitigation of the possibly life-threatening sequelae of inflammatory cardiomyopathy [157].

It is imperative to exercise caution in certain domains to facilitate enhanced comprehension and informed future decision-making. A comprehensive understanding of the influence of genetic factors on the propensity to develop immune-mediated diseases in patients with inflammatory cardiomyopathy is essential. This encompasses an in-depth analysis of the role of HLA genotyping in disease management strategies. Furthermore, there is a need to pursue advancements in our knowledge of the triggers of autoimmunity, including the impact of viruses, medications and other environmentally derived factors.

Further refinement is required in the definition of potential humoral predictors (e.g., specific autoantibody characteristics, pathogenic immunoglobulin class and IgG subclass, autoantibody titre and new relevant autoantigens) and cellular predictors (e.g., immune phenotype of circulating T cells, in particular TH1). It has been established as a priority to enhance the delineation of potential humoral predictors, encompassing distinct autoantibody specificities, pathogenic immunoglobulin classes and IgG subclasses, autoantibody titres and novel relevant autoantigens. In order to further advance knowledge in this field, the immune phenotype of circulating T cells, specifically those belonging to the TH1, TH17 and Treg cell categories, in addition to myocardium-infiltrating cells, comprising T cells, B cells and myeloid cells, is required to be thoroughly characterised. This comprehensive analysis will facilitate more precise understanding of risk of progression to heart failure, mortality, or heart transplantation as well as spontaneous or immunosuppressive therapy-induced recovery in patients suffering from inflammatory cardiomyopathy.

**Figure 2:**
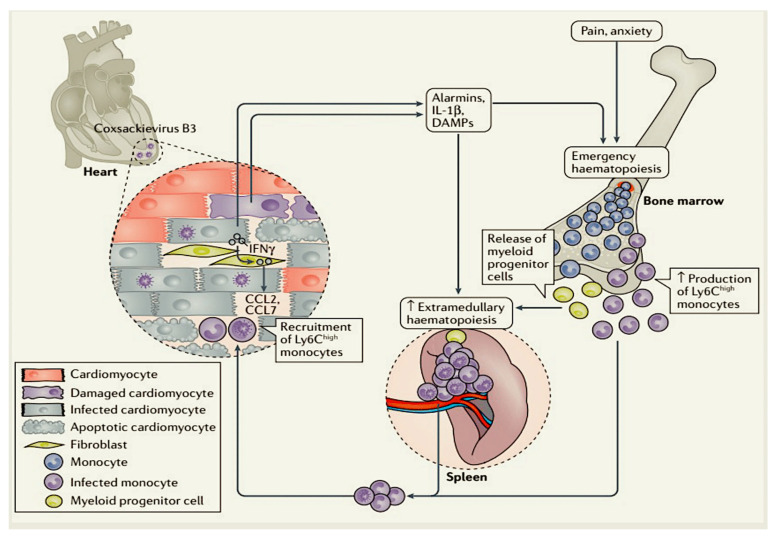
Illustration of cardiosplenic interface in coxsackievirus B3-induced myocarditis. Coxsackievirus B3 infection of cardiomyocytes causes cell death and the release of IL-1β and DAMPs, which trigger the recruitment and activation of innate immune cells. Pain, anxiety and danger signals trigger emergency blood cell production in the bone marrow. Myeloid progenitors then go to the spleen, where new monocytes are made to replace those used up in the damaged heart. Inflammation caused by IFNγ production in infected cardiomyocytes leads to increased CCL2 and CCL7 chemokine production, attracting monocytes to the heart. As the spleen is a target organ of Coxsackievirus B3 and monocytes are target cells of Coxsackievirus B3, the recruited Ly6Chigh monocytes could be infected with Coxsackievirus B3, thereby transporting the virus to the heart and further contributing to viral infection. Activation of the innate immune system in the heart is beneficial for its antiviral effects, but excessive or prolonged activation can lead to inflammation that triggers heart damage and remodelling, causing cardiac dysfunction. License Number 5960170460016; License date 1 February 2025.

Further assessment is required to determine the potential role of inflammatory and pro-fibrotic cytokines (e.g., IL-1, IL-6, IL-17, IL-23 and TGFβ) present at both the peripheral and myocardial levels. These assessments should be made with a view to patient risk stratification. Furthermore, it is necessary to determine the effect of gut microbiome modulation on the course of inflammatory cardiomyopathy. Finally, it is essential to establish a method for distinguishing a beneficial immune reaction that is designed to clear a pathogen, from a pathogenic autoimmune reaction Figure 2.

## 3. General Concept of Diagnosing and Prognosis

### 3.1. Clinical Settings

The cardinal symptoms and indications that typify the eruption of acute myocarditis in clinical subjects encompass thoracic discomfort, shortness of breath, weariness, tachycardia, loss of consciousness and cardiogenic shock [10]. Moreover, acute myocarditis has the capacity to manifest as sudden cardiac death, constituting about 10% of sudden cardiac deaths in young subjects (under 35 years of age) [158]. As demonstrated in the findings of a multicentre study, the initial presentations of acute myocarditis can vary significantly in their manifestation. Specifically, the study revealed that up to 80% of patients exhibited prodromal symptoms, including fever, gastrointestinal disorders, and influenza-like symptoms, in the weeks preceding the onset of the acute phase [7]. Notably, the study also identified that the initial presentations of complicated myocarditis, characterised by a left ventricular ejection fraction (LVEF) of less than 50% on initial echocardiography, sustained ventricular tachycardia, or haemodynamic instability, were associated with a more severe clinical course. In patients exhibiting maintained LVEF, the evaluation of late gadolinium enhancement (LGE) distribution patterns as depicted on cardiac magnetic resonance imaging (MRI) has been demonstrated to enhance prognostic assessment [159,160]. In fulminant myocarditis, a particular type of heart inflammation, the histological subtypes of the disease that cause heart failure, such as giant-cell and eosinophilic myocarditis, have been found to be a significant predictor of mortality [161].

In conclusion, given the importance of timely diagnosis, it is crucial to define the time of cardiac symptom onset, as inflammatory cardiomyopathy can present as the initial manifestation in patients with HF symptoms, potentially resulting from a delayed diagnosis of acute myocarditis. The presence of mild rises in troponin concentrations, which are disproportionate to the severity of the LVEF impairment and associated with a dilated left ventricle at the time of presentation, is indicative of a diagnosis of inflammation-induced cardiomyopathy as opposed to acute myocarditis [161]. In patients exhibiting signs of an underlying inflammatory response within the myocardium, the majority of cases present with a haemodynamic stability that can be attributed to a progressive and often unidentified weakening of the left ventricular systolic function and subsequent remodelling. However, the current literature indicates that the recuperation probability for patients afflicted with myocarditis that has undergone complications stands at a mere 50% [162]. Presently, there exists an absence of recognised clinical indicators capable of accurately predicting the prognosis of patients diagnosed with inflammatory cardiomyopathy.

It is vital to understand how acute myocarditis can lead to chronic inflammatory cardiomyopathy in patients, a topic that has not been widely studied [163]. Furthermore, the investigation should ascertain whether chronic inflammation, a specific infection, or both, within the myocardium is instrumental in this progression, or whether alternative factors are involved. The true incidence of this transition within adult populations should also be ascertained.

It’s also important to understand the true incidence of this change in adult patients. Clinical research tools must be developed, including registries that include diverse patient populations. Registries identify low-risk and high-risk patients. The accuracy of prognostic assessments must be substantiated through various methodologies. One crucial metric is the distinction between uncomplicated and complicated presentations at time of hospitalization; secondly, data from immunohistological [66] or viral analysis [13,146] of EMB samples improves prognostication.

The following research aims have been proposed: firstly, to enhance the accuracy of EMB-based diagnosis and to identify novel and minimally invasive approaches for the diagnosis of myocarditis. Secondly, to develop a multimarker strategy incorporating cardiac MRI to enable independent prognosis, not reliant upon LVEF; thirdly, to identify distinctive biomarkers to facilitate diagnosis and therapeutic decision-making.

### 3.2. Immaging Diagnostics

At present, cardiac magnetic resonance imaging (MRI) represents the non-invasive criterion of choice for the diagnosis of myocarditis (class I recommendation, level of evidence C in the 2012 ESC guidelines) [164]. In 2018, the addition of T2-weighted cardiac MRI to the pre-existing Lake Louise criteria (LLC) for the diagnosis of myocarditis was recommended by consensus guidelines [165]. The 2018 LLC demonstrated superior diagnostic efficacy in comparison to the conventional standards, attributable to enhanced sensitivity [166]. Furthermore, T1 and T2 mapping techniques have contributed additional diagnostic insights and enhanced the precision of cardiac MRI [167,168,169,170,171,172]. Furthermore, the employment of parametric cardiac MRI modalities, such as T1 and T2 mapping, has been demonstrated to enable the quantitative evaluation of inflammatory processes or diffuse myocardial fibrosis. However, it has been observed that the diagnostic accuracy of cardiac MRI may fluctuate in accordance with the clinical manifestation and the extent of cell necrosis in individuals diagnosed with endomyocardial sampling-confirmed instances of acute myocarditis [173]. The diagnostic sensitivity of cardiac MRI has been documented to be notably high in cases of infarct-like presentations, moderate in cases of cardiomyopathy-like presentations, and extremely low in cases of arrhythmia presentations [173]. Moreover, cardiac MRI is unable to establish the type of myocarditis (i.e., the precise immune cell accumulation and the associated causative factors). In conventional clinical practice, numerous subjects exhibit marginally elevated T1 and T2 mapping metrics, which may result in a misdiagnosis of myocarditis. The diagnostic and prognostic significance of these inconclusive results remains to be elucidated.

Cardiac MRI facilitates quantitative assessment of myocardial tissue deformation (strain) through the utilisation of advanced computational methodologies. This methodology encompasses the analysis of existing cine images or the acquisition of specialised sequences, such as displacement encoding with stimulated echoes (DENSE) MRI or fast strain-encoded MRI. The evaluation of strain can be performed in various segments of the myocardium, including the right ventricle and left atrium [174]. Despite the elapse of time since the initial myocardial inflammation, patients may still present with clinical symptoms of dyspnoea. The use of strain analytics, utilising techniques such as echocardiography or cardiac MRI, has emerged as a promising method for the detection of diastolic dysfunction [159,160,175]. However, the literature on the integration of diverse cardiac MRI metrics and their enhanced diagnostic and long-term prognostic capabilities in patients with myocardial inflammation remains limited. Moreover, existing guidelines remain contingent upon conventional assessment of cardiac MRI parameters, thus neglecting the potential benefits afforded by advanced cardiac MRI metrics, such as those afforded by the analysis of strain. Furthermore, there is a necessity to validate cardiac MRI pathways for acute or chronic myocarditis, with a focus on the varying magnetic flux densities (1.5 T and 3.0 T) and for MRI scanners from disparate manufacturers. The validation of these protocols is imperative, and the demonstration of their prognostic value in substantial, multicentre trials is essential to establish the foundation for guideline directives. Furthermore, cardiac MRI has been identified as a highly effective therapeutic monitoring modality in selected patients [176]

Nevertheless, data concerning the optimal scheduling of subsequent cardiac MRI procedures for therapeutic monitoring in patients with myocardial inflammation are currently limited. Despite the well-documented benefits of cardiac MRI in diagnosing acute myocardial inflammation, the technology is underutilised in clinical practice due to its limited availability [177,178,179]. Potential benefits of such a strategy include using mobile cardiac MRI units in collaboration with expert centres. Training and awareness are crucial to improve use of cardiac MRI for inflammation. Cost is a further factor limiting use of cardiac MRI, despite its cost-effectiveness and value for the healthcare system [180,181,182]. Cardiac MRI is often restricted in cases with myocarditis presenting as acute heart failure of a high grade [4,165]. An EMB-guided approach is recommended for high-degree heart block, symptomatic ventricular tachycardia or shock (Class I recommendation, Level B evidence) [183]. New real-time cardiac MRI protocols are under development for the assessment of anatomy, function and perfusion in these patients [184,185,186]. An EMB-based approach is also needed for patients with disease onset >3 months. The diagnostic benefit of cardiac MRI is low in such cases, e.g., in patients with HF of >3 months’ gestation associated with a history of a large LV.

LGE, its increasing, and focal fibrosis on cardiac MRI can predict risk of hospital admission and adverse CV events in individuals with myocarditis [187,188,189]. LGE may also arise on imaging and should be considered a risk indicator even in patients with myocarditis who appear to be improving clinically [190]. LGE parameters may also help to define the presence of cardiac sarcoidosis, regardless of LVEF [191]. 18F-fluorodeoxyglucose (18F-FDG) uptake indicates increased glucose metabolism, a hallmark of inflammation. 18F-FDG PET is a valuable tool for diagnosing and monitoring treatment response in patients with cardiac sarcoidosis. In selected patients with myocardial sarcoidosis, 18F-FDG PET in addition to cardiac MRI may be useful for additional evidence regarding disease severity [192].

Research is ongoing into new ways to measure pressure and energy in the heart using 4D flow cardiac MRI. This may help to better understand patients with heart failure and control therapy [193]. The clinical value of vasodilator stress cardiac MRI for an accurate evaluation of microvascular disease has been demonstrated. Limited evidence is available on the use of this modality in patients with acute myocardial inflammation. With parametric mapping for quantitative estimation of myocardial strain, diffuse fibrosis and oedema, cardiac MRI data may permit the creation of an objective cardiac MRI-based inflammation score, Table S3 Supplementary Materials.

### 3.3. Endomyocardial Biopsy to Diagnose Myocarditis

**Figure 3:**
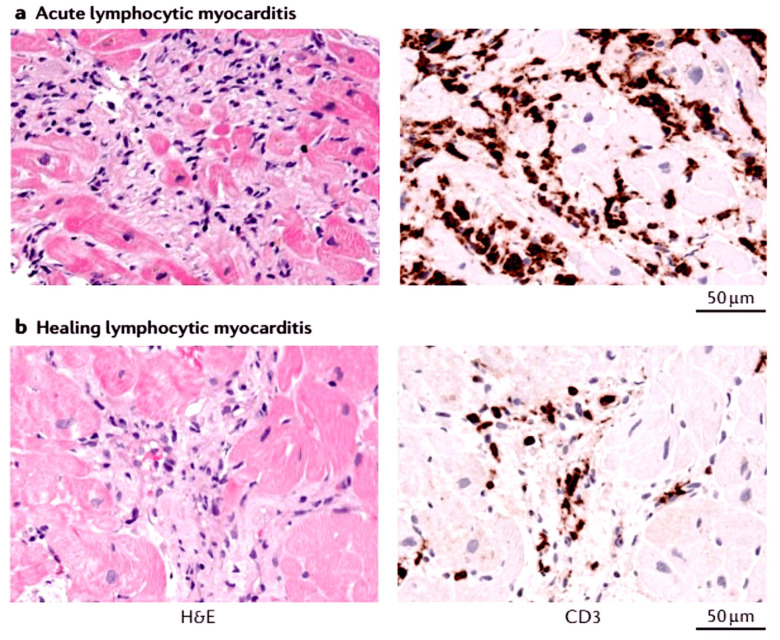
The following illustration depicts the use of an endomyocardial biopsy for the diagnosis of lymphocytic myocarditis. The diagnosis of acute and healing lymphocytic myocarditis is made using histology and immunohistology of endomyocardial biopsy samples. Figure (**a**): Acute lymphocytic myocarditis caused by enterovirus A71 infection. The histology image (left panel) reveals the presence of cardiomyocyte necrosis, a key feature of the condition, as indicated by haematoxylin and eosin (H&E) staining. The immunohistology image (right panel) demonstrates diffuse infiltration of CD3+ T cells, a characteristic feature of the disease, as highlighted by anti-CD3 antibody staining (brown). In figure (**b**), the subject is suffering from healing lymphocytic immune-mediated myocarditis. The histology image (left panel) displays fibrosis, but no cardiomyocyte necrosis, while the immunohistology image (right panel) shows the presence of infiltrated CD3+ T cells. All images have been magnified ×400. License Number 5960170460016; License date 1 February 2025.

It is widely accepted within the medical community that endomyocardial biopsy is the most effective diagnostic method for acute and chronic inflammation of the heart. The Right Ventricular and Left Ventricular Endomyocardial Biopsies (EMBs) have become the standard of care in the clinical assessment of patients requiring a diagnosis of myocarditis. The primary rationale for this acceptance is that biopsies are the sole procedure capable of determining the causative agent responsible for the inflammation of the heart [4,162,194,195,196,197]. A scientific publication by the European Society of Cardiology (ESC) in 2013 proposed a characterisation of cardiac inflammation using immunohistochemistry and viral genome analysis by quantitative PCR (real-time PCR and nested PCR with reverse transcription) for the diagnosis of myocarditis and the determination of appropriate pharmacological treatment plans [4,162]. In addition, immunohistochemistry employing a range of monoclonal and polyclonal antibodies (comprising anti-CD3, anti-CD68 and anti-HLA-DR antibodies) is advocated for the characterisation of the inflammatory infiltrate. In comparison with the histological Dallas criteria, immunohistochemistry has been demonstrated to be more sensitive [198] and has been shown to have prognostic significance [66,194]. Given that cardiac inflammation frequently exhibits an uneven distribution, analysis of a minimum of five or six tissue specimens is recommended in order to minimise the potential for inaccuracy associated with EMB sample extraction [199] Figure 3.

Moreover, as a result of the pivotal role played by many viral infections, the use of two or three EMB specimens is also advised for the identification of viral nucleic acids, thus circumventing the occurrence of false-negative outcomes [200,201]. Notwithstanding this awareness and the concomitant risk of under-diagnosis, clinicians frequently demonstrate a reluctance to obtain in excess of four EMB specimens during a conventional clinical course, a reluctance largely attributable to apprehensions regarding untoward consequences such as ventricle perforation. Consequently, the implementation of integrated methodologies encompassing EMB and imaging or electroanatomical mapping has been posited as a potential means to surmount this predicament. T2-mapping cardiac MRI has the potential to assist healthcare professionals in determining which patients may derive benefit from proceeding with EMB for the purpose of informed treatment decisions [202,203] (see Table 1). In fact, there is a growing body of literature to support the notion that the employment of 3D electroanatomical voltage mapping [204,205,206,207], cardiac MRI [203], or 18F-FDG PET [208] to provide a framework for EMB is beneficial in enhancing the sensitivity and specificity of the standard EMB modality. This is achieved by minimising artefactual errors and facilitating a more profound comprehension of diverse (local) pathological conditions [209].

Comprehensive gene expression profiles of the heart in the inflammatory state can currently only be obtained from advanced molecular analyses of heart tissue specimens, primarily derived from endomyocardial biopsy. Gene expression profiling may assist in the accurate diagnosis of idiopathic giant-cell myocarditis and cardiac sarcoidosis [210]. In order to study the role of omics technologies, such as genomics, epigenomics, proteomics and metabolomics, in diagnosis and pharmaceutical development, it is necessary to correlate these with state-of-the-art methods including histology, immunohistochemistry and molecular virology. Targeted biopsies of the inflamed heart are required for this purpose. A pioneering study utilising globally focused proteome screening has demonstrated a correlation between the presence of inflamed heart conditions and alterations in the extracellular matrix, along with a decline in proteins implicated in carbohydrate metabolism, the tricarboxylic acid cycle, and oxidative phosphorylation [211]. Employing mass spectrometry analysis of EMB specimens, a method that facilitates region-specific evaluation of protein profiles, enabled the segregation of patients into distinct clusters based on their response to cardiac inflammation [212].

The combination of patient epigenetic profiles with other genetic strategies, including next-generation sequencing (NGS), has the potential to illuminate the intricate gene networks that underpin myocarditis in subjects who subsequently progress to HF [213]. The utilisation of NGS has resulted in the recognition of an escalating array of gene variants and mutations that are correlated with an augmented risk of heart disorders, including DCM. The increased use of genome sequencing is likely to result in the detection of additional high-risk gene mutations, thereby enhancing the clinical decision-making process and providing insights into the pathogenesis of inflammation-induced cardiomyopathy. The effective incorporation of these omics techniques into existing diagnostic algorithms is expected to contribute to a more sensitive, specific and cost-effective approach for the personalized treatment of patients with inflammation-induced cardiomyopathy [214]. It is further asserted that the application of NGS will serve to enhance the identification of hitherto uncharacterised pathogenic agents of a cardiotropic nature, including DNA and RNA virus strains, in instances of myocardial inflammation [215].

The standardization of a range of immunohistochemistry markers and assays for the analysis of EMB specimens is of paramount importance. This process involves the selection of formalin-fixed tissue over frozen tissue sections and the determination of the most appropriate antibodies for a specific objective [198]. Firstly, it is imperative to ascertain that the number of biopsies necessary for histological, immunohistological, and viral diagnostic procedures is standardised. Secondly, there is a need to establish a more comprehensive classification system for specific immune cell subtypes (e.g., Treg cells, TH17 cells, and pro-inflammatory and anti-inflammatory monocyte and macrophage subsets) implicated in myocardial inflammation and inflammatory cardiomyopathy.

### 3.4. The Process of Genetic Testing and the Use of Biomarkers

#### 3.4.1. Genetic Testing

Although monogenic familial occurrences of acute heart inflammation or chronic inflammatory heart diseases are uncommon, arrhythmogenic cardiomyopathies, specifically those resulting from heterozygous pathogenic variants in DSP, have been linked to heightened cardiac inflammation and a clinical manifestation of acute myocarditis, accompanied by elevated plasma troponin concentrations, in addition to characteristic cardiac MRI or PET-CT scan alterations [216,217,218,219,220]. The presence of specific genetic variations, or variants, in genes linked to hereditary arrhythmogenic cardiomyopathies (ACM) has been observed to be more prevalent in children diagnosed with acute myocarditis than in those considered to be healthy. This observation has been noted to be particularly pronounced in homozygous, but not heterozygous, carriers of these rare variants [221]. The underlying mechanisms that trigger the heightened cardiac inflammation observed in ACM cases remain to be fully elucidated. Notwithstanding, the implementation of genetic screening is strongly advocated in all familial forms of myocarditis, in familial cardiomyopathy, and when indications of arrhythmogenic cardiomyopathy become evident through diagnostic imagery or electrophysiological assessment.

#### 3.4.2. The Role of Microrna Profiles in EMB Specimens and Blood

Genes can be affected by environmental factors that increase the risk of heart inflammation. MicroRNAs (miRNAs) regulate the immune response in the heart [222,223,224,225,226]. Checking microRNA levels in heart muscle samples can help distinguish between types of heart inflammation [227,228]. For instance, 107 types of microRNA were discovered to be present in higher or lower amounts in samples from the right side of the heart (RV) of patients with acute viral myocarditis compared to samples from people without heart problems [229]. Also, eight types of microRNA were found to be present in higher amounts in samples from patients with myocarditis who also had a virus called CVB3. This was compared to samples from patients who had the virus cleared from their body without heart problems [230]. In a study of heart samples from mice with a condition called Trypanosoma cruzi-induced myocarditis, 113 of 641 types of molecules (miRNAs) were found to be differently expressed than in control mice [231]. A study looking at heart-related, fibrosis-related and leukocyte-related molecules in blood found that only molecules related to heart muscle injury (including miR-208 and miR-499) were increased in patients with acute myocarditis compared with people without the condition [232,233]. However, these levels of heart muscle injury are not specific, because they are also increased in patients with heart attacks caused by blood clots or high blood pressure. It is interesting to note that the levels of certain molecules (miRNAs) in the blood are linked to inflammation, including miR-21, miR-146b and miR-155, were not increased in patients with acute myocarditis compared with control individuals even though leukocyte counts were elevated [232,234], which possibly reflects the absence of miRNA release by inflammatory cells.

We still do not understand the connection between circulating and tissue miRNAs very well and this needs to be looked into further. Future research should look at different types of cells (e.g., white blood cells and heart muscle tissue) and samples (tissue removed during a procedure called a biopsy) to better understand how disease affects the body. This could lead to new ways to spot differences between various forms of heart inflammation, as well as to tell the difference between heart inflammation and a lack of blood flow to the heart, which is a big medical problem that we still have not found a solution to.

#### 3.4.3. Novel Diagnostic Procedure: EMB Transcriptome—Based Biomarker

Recent studies have demonstrated that, in conjunction with microRNAs, a range of mRNAs can serve as highly effective diagnostic tools for identifying lymphocytic myocarditis [235]. A microarray-based transcriptome biomarker has been shown to possess 100% sensitivity and specificity in the diagnosis of myocarditis in EMB specimens [236]. The most streamlined transcriptomic profile revealed a high degree of significance for immune markers, with particular emphasis on various constituents of the TLR family [236]. The application of this transcriptomic-based biomarker has proven effective in the identification of lymphocytic myocarditis and active cardiac inflammatory conditions in EMB specimens from patients diagnosed with rheumatic disease or peripartum cardiomyopathy [235,236]. Further research is currently underway to ascertain the efficacy of this approach for liquid biopsy [236].

#### 3.4.4. Blood Related Biomarkers

In addition to the aforementioned EMB-based bioindicators, there is growing evidence that a number of blood biomarkers, including high-sensitivity C-reactive protein, N-terminal pro-B-type natriuretic peptide, troponin T and soluble IL-1 receptor-like 1 (IL1RL1; also known as ST2), have been the focus of research in the context of myocarditis and inflammatory cardiomyopathy [4,34,82]. Men under 50 with suspected heart problems and confirmed by EMB have higher levels of a protein called ST2 in their blood. This is linked to a higher risk of severe heart failure, as judged by the New York Heart Association classification of heart failure [34].

This shows that measuring soluble ST2 can be used to predict the risk of heart failure in men, and that it is important to look at inflammation-related proteins like soluble ST2 in different ways, depending on the person’s age and sex. This also highlights the need for biomarkers that can predict the risk of heart failure in women with myocarditis [34]. The presence of specific blood biomarkers capable of informing diagnoses in patients with suspected myocarditis has yet to be determined. Furthermore, the same applies to the ability of such biomarkers to determine the existence or non-existence of active myocardial inflammatory processes [4,82]. Nevertheless, the results of a number of recent studies suggest that plasma concentrations of the S100A8-S100A9 heterodimer, which are primarily secreted by monocytes and neutrophils, may serve as a reliable indicator of disease activity in cardiac tissue samples from patients with recent-onset myocarditis [86]. Moreover, initial findings indicate that S100A8-S100A9 could function as a diagnostic and therapy-tracking biomarker in subjects with presumed acute heart inflammation within 30 days of symptom onset [86].

It has been observed that patients suffering from autoimmune myocarditis [237] or idiopathic DCM [238] exhibit a decrease in circulating Treg cells, concomitant with an augmented response of circulating TH17 cells, when compared to healthy individuals. Consequently, the quantification of blood Treg cell and TH17 cell populations in these patients might be advantageous in informing therapeutic decisions and monitoring therapy outcomes, particularly in light of the existence of therapeutic interventions capable of enhancing the Treg cell to TH17 cell ratio. Inflammatory cardiomyopathy has been demonstrated to be precipitated, at least a proportionately minor level, by the initiation of heart-specific CD4+ T cells, which are induced by myosin peptide mimics that are derived from *Bacteroides thetaiotaomicron*, an intestinal commensal bacterium. This observation indicates that the assessment of IgG antibodies that are directed towards this intestinal variety could have the potential to facilitate the decision-making process in relation to antibiotic therapeutic intervention [157].

In the future, research may be directed towards the real-time monitoring of disease evolution through the investigation of liquid biopsies that could provide information about the differences in cardiac tissue by looking at cell types (e.g., immune cells) and their products at designated intervals. This could help to see how diseases change over time. The use of NGS platforms to assess blood samples can facilitate the identification of novel biomarkers linked to inflammatory heart diseases such as DNA methylation, histone modification and microRNA makers [239]. Another approach involves the analysis of the proteome of exosome-rich serum to identify myocarditis subtypes and develop predictive and prognostic biomarkers [240].

The existing knowledge gap and future directions for research in this field should include a deeper understanding of the reasons behind the observed associations between variants in DSP and arrhythmogenic cardiomyopathies. These associations have been demonstrated in the context of cardiac MRI scans, which reveal the presence or absence of myocarditis. To further advance the field, it is necessary to develop tools that are directly focused on myocarditis-specific blood biomarkers. These biomarkers should be able to inform the diagnosis of patients with suspected myocarditis and help to determine the presence or absence of active myocarditis. Furthermore, there is a need to develop markers that can be used for therapy monitoring. In addition, there are several further steps that should be taken in order to achieve greater clarity on this matter. Firstly, it is necessary to identify biomarkers that predict the risk of HF in women with myocarditis. Secondly, the reason for the lack of correlation between microRNA levels in blood and EMB samples must be established. Thirdly, it is essential to determine whether potential markers developed on the basis of circulating cells would be more sensitive and specific in diagnosing and discriminating myocarditis from other causes than markers developed on the basis of EMB samples.

## 4. Treating Patients

### 4.1. Treating Heart Failure and Abnormal Heart Rhythms

In the treatment of patients diagnosed with myocarditis and reduced LVEF, the guidelines for the management of HF are to be followed, with optimal medical care being provided [241]. However, it should be noted that a significant proportion of patients diagnosed with myocardial inflammation have LVEF that is not reduced. The efficacy of early initiation of treatment with inhibitors of the renin–angiotensin–aldosterone system or with β-blockers in reducing inflammation, adverse remodelling and scar formation in these patients is unclear. It is particularly uncertain whether the risk of developing arrhythmias in individuals with myocardial inflammation is influenced by LVEF [242].

In patients suffering from myocarditis, serious brady and tachyarrhythmias have the potential to materialise at any point during the course of the disease, with the possibility of ultimately resulting in sudden cardiac arrest [158,243]. The prevalence of ventricular arrhythmias (VA) has been documented as 29% in cases of giant-cell myocarditis [244] and 55% in instances of cardiac sarcoidosis [245]. In contrast, supraventricular arrhythmias manifest with greater frequency than VA in the context of myocardial inflammation, exhibiting variability in prevalence contingent on the specific myocarditis type [246]. Atrioventricular block is observed with a lower frequency in cases of acute or fulminant myocarditis compared to instances of cardiac sarcoidosis, with a comparatively low manifestation of the condition in individuals diagnosed with giant-cell myocarditis [240] (9). The occurrence of cardiac electrical conductance abnormalities exhibits a decline from cases of giant-cell myocarditis to those of eosinophilic myocarditis, and subsequently to instances of myocarditis of a lymphocytic nature [247]. This observation signifies a pronounced clinical necessity to discern individuals afflicted with myocarditis who are predisposed to arrhythmias, a discernment that must supersede the assessment of left ventricular ejection fraction and the presence of late gadolinium enhancement. A multitude of pathological processes have been postulated to provide a rationale for the occurrence of various arrhythmias observed in patients with acute myocarditis. These include electrical dysfunction resulting from direct cytopathic effects; ischemia due to coronary microvascular or macrovascular disease; dysfunction of gap junctions; abnormal calcium handling; and involvement of the cardiac conduction system. The potential for fatal sudden cardiac arrest in patients with acute myocarditis has been observed to be independent of the severity of myocardial inflammation, persisting even after the resolution of the acute phase of myocarditis [21]. The development of ventricular arrhythmias, potentially attributable to scarring, has been documented in subjects with resolved myocarditis, manifesting a form of monocentric ventricular tachycardia [248,249].

Following the occurrence of inflammation, scarring related to ventricular arrhythmias manifests in regions of myocardial fibrosis. These regions are identified as low-voltage areas on electroanatomical voltage mapping or as late gadolinium enhancement on cardiac magnetic resonance imaging. While systolic dysfunction is a prevalent discovery in subjects exhibiting VA, the development of an arrhythmogenic scar is a possibility in subjects with maintained LVEF [21]. EMB is advocated for the purpose of identifying patients with underlying myocardial inflammation who also exhibit signs of VA, given the heightened risk of VA in patients with inflammation present in EMB samples. The presence of viral deoxyribonucleic acids in specimens obtained from the endomyocardial biopsy procedure may serve as an indicator of an elevated risk of ventricular arrhythmias and subsequent myocardial injury characterised by progressive electrical conduction abnormalities. In a mouse study of CVB3-induced myocarditis, the effects of CVB3 infection on cardiac ion channels KCNQ1, hERG1 and Cav1.2 were found to be modulated over time. This suggests an underlying mechanism for the increased risk of arrhythmias in enteroviral myocardial inflammation [250]. In the context of diagnosing cardiac inflammation in patients with VA, endomyocardial biopsy has been observed to exhibit a high degree of variability after specimens recover, particularly in individuals with focal myocarditis and those with cardiac sarcoidosis. To address this challenge, electroanatomical voltage mapping has emerged as a valuable technique, enabling the precise targeting of the bioptome (the instrument employed for the acquisition of EMB fragments) to regions exhibiting an amplitude of less than 0.5 mV and a fractionated electrogram signal. This approach offers a more accurate and reliable method for sample acquisition, enhancing the diagnostic precision of EMB in the assessment of cardiac inflammation [204,205,206,207].

The standard management strategy for patients with acute myocarditis involves the administration of antiarrhythmic medications. However, the efficacy of this treatment modality remains to be substantiated by rigorous clinical trials. The optimal timing for the evaluation of cardiac device implantation for the management of VA remains unclear. It is generally recommended that this evaluation be conducted after the resolution of reversible acute myocarditis, which typically occurs 3–6 months after the commencement of the acute phase [251]. However, the optimal interval for the insertion of an implantable cardioverter–defibrillator (ICD) continues to be a subject of debate. In some cases, the initiation of temporary pacemaking might be necessary upon presentation, but the decision to proceed with chronic pacing is typically preceded by a prolonged phase of monitoring, in addition to histological examination of extracted specimens and a thorough assessment of the progression of the illness. Recent studies have indicated that avoiding early de novo ICD implantation in cases of depressed LVEF is advisable. In situations where patients are deemed to be at high risk of sudden cardiac death, the utilisation of a wearable cardioverter–defibrillator (LifeVest, ZOLL) is recommended [252]. This recommendation is also applicable to patients suffering from lymphocytic myocarditis, as well as those experiencing myocarditis in conjunction with VA during the acute stages of the illness [253]. Nevertheless, the optimal timing of wearable cardioverter–defibrillator use remains to be determined through prospective investigation. Patients diagnosed with giant-cell myocarditis and those with a heart transplant who have a heart transplant-free life expectancy of >1 year should receive an implantable cardioverter-defibrillator. The risk stratification of patients afflicted with cardiac sarcoidosis appears to be largely independent of LVEF, underscoring the necessity for the identification of LVEF-independent markers.

### 4.2. Pharmaceutical and Biological Products

A number of therapeutic interventions have been examined in particular patient groups suffering from inflammation of the heart muscle. These interventions have been developed on the basis of the detection of viral genome (virus type and viral load) and immune cell infiltrates, as defined by the EMB.

#### 4.2.1. The Heart Muscle Has Been Inflamed, Without Being Infected with a Virus

The utilisation of immunosuppressive therapeutic agents, such as prednisone and azathioprine, has been demonstrated to enhance cardiac function in cases of virus-negative chronic inflammatory cardiomyopathy, as evidenced by studies and registries of EMB specimens from affected individuals [143,144,254,255,256]. This observation stands in contradistinction to the findings of preceding investigations conducted on patients with acute cardiomyopathy, wherein the presence of viral pathogens was not a subject of inquiry.

A single-centre study of an observational nature was conducted, which revealed that 53% of patients diagnosed with inflammatory cardiomyopathy who exhibited an unresponsive nature to steroid-based therapeutic intervention were found to possess CD20+ B cells in samples derived from endomyocardial biopsies [130]. The study revealed that in a group of six patients with virus-negative inflammatory cardiomyopathy who also exhibited CD20+ B-cell-positive EMB results, treatment with rituximab, a chimeric monoclonal antibody directed against the pan-B-cell surface molecule CD20, resulted in improvements in cardiac function and alleviation of symptoms related to heart failure when compared to the baseline status of the patients [130]. This suggests that rituximab therapy may have beneficial effects in patients diagnosed with these particular conditions. Conversely, therapeutic interventions for patients diagnosed with virus-negative or autoimmune inflammatory cardiomyopathy encompass the administration of steroids in conjunction with cyclosporine [257] or mycophenolate mofetil [258], or immunoadsorption followed by intravenous immunoglobulin (IVIG) administration (referred to as immunoadsorption-IVIG) [259,260,261]. Non-specific immunoadsorption has been used to remove circulating antibodies with success in the therapeutic management of multiple autoimmune diseases [262,263]. A body of pilot studies indicates that immunoadsorption is effective [259]. IVIG improves heart function in patients with DCM and reduces inflammation [264]. The treatment may work for other autoimmune conditions, but further research is needed. A large, placebo-controlled multicentre study to investigate the effects of immunoadsorption–IVIG on LV function in patients with DCM or inflammatory cardiomyopathy is underway [265]. This study will provide further data to support the hypothesis. The randomisation step of the trial was finalized in 2019 [265]. As an alternative to the previously mentioned immunoadsorption procedural approach, the intravenous administration of reduced molecular weight soluble substances that target and neutralise autoantibodies against the β1-adrenergic receptor is being investigated [266]. These antibody-targeting strategies are not contingent upon the existence of cardiac inflammatory responses, as illustrated in central picture.

#### 4.2.2. Patient Diagnosed with Viral Positive Inflammatory Cardiomyopathy

Distinctions between virus-induced active myocarditis (e.g., adenoviruses or enteroviruses) and virus-associated myocarditis (where the viral genome is detected in samples obtained by endomyocardial biopsy but it is unclear if the virus is a bystander, e.g., due to latent infections with herpes viruses or B19V) are significant (central picture). To address this knowledge gap, further research is necessary to establish the effectiveness of therapies targeting viral activity in acute viral myocarditis through randomised clinical trials. One such trial is the phase II BICC trial [267] which explored the impact of immuno-modulation using IFNβ therapy on viral eradication in patients with inflammatory cardiomyopathy and myocardial viral persistence (including adenoviruses, enteroviruses, and B19V). In trial cohorts with enterovirus-positive myocarditis or adenovirus-positive myocarditis (as determined by EMB), the administration of IFNβ26,260 resulted in viral eradication. However, IFNβ therapy did not correlate with viral DNA clearance in patients with B19V-positive myocarditis. Conversely, the antiviral drugs pocapavir and pleconaril, along with IVIG treatment, have been found to be remarkably successful in neonatal cases of enteroviral myocardial inflammation [23,24,25]. The utilisation of anti-herpesvirus medications is a potential approach for patients with latently infected Epstein-Barr virus, cytomegalovirus, or human herpesvirus 6, with the objective of reducing viral copy quantities [268]. The investigation into the potentiality of a combination of antiviral and immunosuppressive medications as a therapeutic option for selected patients with virus-positive inflammatory cardiomyopathy, contingent on the disease’s stage, is imperative.

Intravenous immunoglobulin is frequently administered to patients with severe B19V viraemia and associated clinical manifestations. Recent studies have focused on the development of novel antiviral medications to combat B19V infections [62]. with several compounds under investigation including synthetic nucleotide analogues, such as cidofovir and brincidofovir, flavonoid molecules, and hydroxyurea. Nevertheless, there is currently a paucity of therapeutic options for the management of B19V-associated inflammatory cardiomyopathy. The prevailing perspective is that no therapeutic intervention is required if low B19V copy numbers are detected in heart tissue specimens in the absence of heart inflammatory changes [4]. Evidence from a series of preliminary observational studies suggests that immuno-suppressive medication can have a positive impact on individuals with low concentrations of B19V DNA in the heart muscle and with cardiac inflammatory responses (CaPACITY programme) [269]. Furthermore, the antiviral drug telbivudine, which possesses immunomodulatory properties, has been observed to be effective in patients with B19V RNA positivity [64,162]. Nevertheless, the validation of these findings through placebo-controlled clinical trials is imperative. It is noteworthy that immunoadsorption–IVIG has been demonstrated to be both innocuous and efficacious in ameliorating clinical symptoms in patients diagnosed with virus-positive inflammatory cardiomyopathy, irrespective of the detection of B19V or HHV6 in EMB specimens [270]. Conversely, a study that did not evaluate cardiac viral load demonstrated that IVIG did not show any favourable impacts in subjects with DCM [271]. However, data obtained from medical records suggests a positive correlation between the administration of IVIG and clinical enhancement in cases of myocarditis associated with infection by the B19V virus [272]. The study noted that IVIG resulted in a reduction in cardiac inflammatory responses, while the elimination of cardiac B19V as measured by EMB specimens appeared to be constrained. In treating cases of HIV-associated, HCV-associated, or influenza-associated myocarditis or inflammatory cardiomyopathy, the treatment paradigm involves the administration of established antiviral medications (see Table 1). This includes the provision of antiretroviral medication for patients diagnosed with HIV-associated myocarditis [273], in conjunction with a combination of ombitasvir, paritaprevir, ritonavir and dasabuvir for patients with HCV-associated myocarditis [79], and neuraminidase inhibitors (peramivir and zanamivir) for patients with myocarditis associated with infection by influenza [274,275].

In the treatment of patients diagnosed with the SARS-CoV-2 infection, several investigational antiviral regimens are currently in development. These regimens encompass strategies designed to impede viral entry into the host cell, such as the administration of chloroquine, hydroxychloroquine, and camostat mesyl. Other strategies include the administration of protease inhibitors (lopinavir–ritonavir and darunavir), RNA polymerase inhibitors (remdesivir), and anti-cytokine agents (such as IL-6 receptor antagonists and IL-1β inhibitors) [276].

## 5. New Types of Administered Medications

In the absence of a response to guideline-directed neurohormonal inhibitor therapy and haemodynamic support, it is recommended that individuals be considered for alternative therapeutic interventions. Such interventions may take the form of therapies that either target one or more of the effector arms of the immune response or that encourage regulatory mechanisms within the immune response. The results of seminal clinical studies suggest that a plethora of signalling molecules may be selectively engaged in subjects diagnosed with myocardial inflammation or inflammatory myocardial disease, emphasising DCM. It is important to note that contemporary clinical trials are being conducted with the objective of utilising the knowledge gained from prior investigations of elevated TNF inhibitor treatment in patients diagnosed with systolic heart failure. These earlier trials did not demonstrate enhancements in patient prognoses [277]. The present generation of therapeutic agents is characterised by a systematic approach to the personalisation of targeted therapies. This development is motivated by the need to minimise adverse effects and maximise the probability of successful outcomes in patients exhibiting particular phenotypes of inflammatory heart disease.

- Aldosterone antagonists

Recent studies have demonstrated that initial mineralocorticoid receptor blockade during the acute phase of CVB3 infection using eplerenone results in pleiotropic responses, including immunomodulatory, anti-oxidative, and anti-apoptotic influences. These effects contribute to the prevention of detrimental cardiac remodelling and dysfunction without exerting an effect on viral load within the heart, as observed in a mouse experimental model of sustained viral myocardial inflammation [278]. The results of this study indicate that eplerenone is a promising option for the therapeutic management of acute myocarditis, particularly in conjunction with HF therapies. However, the prevailing guidelines do not currently encompass the utilisation of aldosterone antagonist therapies for the treatment of acute myocarditis. This underscores the necessity for the rigorous evaluation of this novel therapeutic concept through the framework of clinical investigation.

- Cannabidiol and antagomirs

Myocarditis is the term given to an inflammation of the heart muscle, and experimental studies are underway to determine the efficacy of interventions that primarily target the immune system’s regulatory functions in the treatment of this condition. The therapeutic interventions encompass the administration of cannabidiol [279] and antisense microRNA complements [87], which are designated as antagomirs or anti-miRs. The therapeutic potential of antagomirs, whether administered systemically or locally, lies in their capacity to modulate inflammation or virus replication [87], thus functioning as a therapeutic agent.

- Modulating the gut microbiome

Increasing evidence supports the involvement of the gut microbiome and its related metabolites in the contribution to the downstream inflammatory processes linked to HF [280]. In the same way, a myosin mimetic peptide originating from the gut microbiota has been associated with induced inflammatory heart disease [157]. These discoveries indicate that modulation of the microbiome and its associated metabolic products are promising prevention and treatment modalities for inflammation-related heart conditions.

- Immunotherapies
  ▪ **Soluble anti-CAR antibody.** A pair of investigations centred on a treatment involving an engineered soluble CAR that has been fused to the carboxy terminus of human IgG, a process that has been demonstrated to curtail virus uptake into host cells, with the objective of curtailing the progression of both acute [281] and chronic [282] CVB3-induced myocarditis in murine subjects. Nonetheless, the feasibility of this approach in human subjects remains to be ascertained.
  ▪ **Anti-IL-1β and anti-IL-1 receptor antibodies**. Studies of viral and autoimmune myocarditis in animal models have yielded evidence that lends support to a central role for NRLP3 inflammasome signalling and subsequent IL-1β production in the etiopathogenesis of myocardial inflammation [283] (68). The administration of an anti-mouse IL-1 receptor antibody at various phases of enteroviral infection has been demonstrated to impede the progression of chronic viral myocarditis in murine models by attenuating inflammation, interstitial fibrosis, and deleterious cardiac remodelling [283]. There is supporting evidence from one clinical study [284] and a number of case reports [285,286] for the use of an anti-IL-1β monoclonal antibody in the management of patients with a recurrence of pericarditis. The underway ARAMIS [287] and RHAPSODY [288] trials have been developed to evaluate the effectiveness of IL-1β-blocking medications in individuals diagnosed with myocarditis and concomitant pericarditis.
  ▪ **Anti-IL-17 antibody**. The present body of research indicates that heightened IL-17-related responses and the onset of profibrotic mechanisms have been correlated with an elevated risk of mortality in murine models of CVB3-induced myocarditis [289] and with a diminished rate of restorative functional recuperation in human myocarditis cases [124]. It has been demonstrated that TH17 cells can induce the development of dilated cardiomyopathy in murine models [118]. Conversely, Treg cells have been observed to offer a protective effect against myocarditis by moderating inflammatory responses [96,119]. A clinical trial involving the administration of secukinumab, a monoclonal antibody targeting IL-17, has been put forward as a potential therapeutic approach.
  ▪ **Cell-based therapies**. The clinical implementation of Treg cells [290] and the utilisation of IL-2 agonists [291] whose function is to stimulate Treg cell proliferation and to enhance the survival and suppressor function of mature Treg cells [292] represent complementary strategies to elevate the Treg cell to TH17 cell ratio. It has been posited that an alternative cell-based strategy may entail the utilisation of mesenchymal stromal cells, which have been demonstrated to enhance the population of Treg cells [101] and have been observed to exert immunomodulatory and cardioprotective functions in murine models of myocardial inflammation [85,94,293], potentially through the moderation of the cardiosplenic interface. The safety and efficacy of therapy with allogeneic mesenchymal stromal cells has been demonstrated in patients with non-ischaemic DCM in the POSEIDON-DCM trial [294]. The POSEIDON-DCM trial revealed that the LVEF exhibited a notable enhancement following autologous mesenchymal stromal cell therapy, yet this observation was exclusively confined to patients devoid of a pathogenic gene variant associated with DCM. This finding underscores the significance of a patient’s genetic profile in determining their responsiveness to mesenchymal stromal cell therapy [295]. A notable finding in this regard was the significant correlation between this response and a considerable decrease in circulating TNF levels [294], thereby pointing to an immunomodulatory therapeutic effect. In conclusion, this set of observations signifies the potential for cell-based therapy in the management of subjects diagnosed with inflammation-induced cardiomyopathy, thereby underscoring the necessity for further investigation in this area through well-designed clinical trials.

## 6. Mechanical Circulatory Support in Patients with Fulminant Myocarditis

In instances of cardiogenic shock arising from fulminant myocardial inflammation, the administration of parenteral inotropes and short-term mechanical circulatory support (MCS) systems is frequently necessary. The initiation of immunosuppressive therapy does not preclude the use of MCS. The efficacy of various MCS devices in achieving short-term haemodynamic stability has been well-documented, with some studies even suggesting their use as a bridge to transplant in patients with fulminant myocarditis. Notable examples of this include veno-arterial extracorporeal membrane oxygenation (V-A ECMO) [296,297], and intra-aortic balloon pumps (IABPs) [297,298] the percutaneous ventricular assist devices TandemHeart [297] and ProtekDuo [299], and the Impella microaxial flow catheters [297,298,300]. The fundamental distinction between these devices lies in their respective modes of action, particularly with regard to their impact on afterload modulation. The impact of these devices on afterload modification in fulminant myocarditis warrants particular consideration, as an augmentation in peripheral resistance can further stimulate cardiac inflammatory responses due to an escalation in ventricular wall stress. A high flow of V-A ECMO has been linked to higher LV afterload. It has been hypothesised that this may result in an inflammatory response in the heart, which could potentially lead to remodelling and subsequent changes in its shape throughout the course of the disease. IABP and TandemHeart have a limited impact on reducing LV afterload, while intravascular aortic catheter (iVAC) and the even more effective LV Impella microaxial flow catheter systems (Impella CP, 5.0 and 5.5) support blood flow in the body’s periphery along with ventricular emptying [301]. As demonstrated in the extant literature, mechanical load has been observed to activate the cardiac mechano transduction pathway [302]. This pathway has been shown to be linked to adverse cardiac remodelling and the activation of cardiac fibroblasts, which in turn can further promote inflammatory reactions [303]. The available data from LV assist devices in patients with chronic heart failure suggests that mechanical load reduction can result in reverse remodelling involving immunomodulatory processes [304,305]. Preliminary single-centre studies suggest that extended utilisation of microaxial flow catheters over weeks engenders distinctive anti-inflammatory, disease-modifying effects that extend beyond circulatory support, as outlined by the PROPELLA concept [301,306].

In routine clinical scenarios, the selection of MCS device is predominantly dictated by its availability at the centre in question and the extent to which the left or right ventricle is affected, or indeed both ventricles. Patients with primary right ventricular (RV) or biventricular failure are generally treated with an extracorporeal centrifugal flow-based regimen comprising V-A ECMO, iVAC, TandemHeart and ProtekDuo [297]. A review of the literature reveals that analyses of data from experienced centres demonstrate the safety and potential efficacy of combining a V-A ECMO with an IABP, an iVAC (EC-iVAC), or an LV Impella (ECMELLA) for patients diagnosed with fulminant myocarditis [298,307,308]. This combination appears to exceed the benefits of a solitary V-A ECMO strategy. A paucity of data exists pertaining to the efficacy of the RP Impella system and/or its combination with the LV Impella in unloading both venules (BIPELLA-concept) in cases of fulminant myocarditis [306,309]. In the case of patients with mainly LV failure and preserved RV function, the utilisation of the LV Impella system for unloading might prove beneficial [162,300,301,306]. A paucity of data exists pertaining to the efficacy of the RP Impella system and/or its combination with the LV Impella in unloading both venules (BIPELLA-concept) in cases of fulminant myocarditis [306,309,310]. In the case of patients with mainly LV failure and preserved RV function, the utilisation of the LV Impella system for unloading might prove beneficial [162,300,301,306].

Further research is required to elucidate the processes underpinning the anti-inflammatory impact of prolonged LV emptying (PROPELLA concept) [301], particularly in conjunction with percutaneous LV support devices, which have the potential to facilitate recuperation or transition to transplantation. Moreover, the conduction of clinical trials is essential to substantiate the PROPELLA concept, ideally incorporating EMB analysis. Additionally, further investigation is required to ascertain whether pulsatile systems (such as iVAC and IABPs) demonstrate divergent characteristics compared to non-pulsatile systems. Future priorities and research gaps for treating patients in Supplementary Materials Table S4.

## 7. Conclusions

Myocarditis and inflammatory cardiomyopathy were initially delineated during the early 20th century based on the study of Fiedler [311]. Following a series of seminal discoveries involving the EMB technique and the seminal study by Sakakibara and Konno [312] which was corroborated by other research groups [313,314], the advent of PCR and the evolution of cardiac MRI [315,316], and expert opinion statements, has led to a revised definition of myocarditis and inflammatory cardiomyopathy. The most recent substantial review publication dates back to 2013 [4]. The aforementioned sequence of events indicates that the domain of myocarditis and inflammatory cardiomyopathy is a relatively contemporary entry into the field of cardio-science. As the 2020s begin, there are still significant unanswered questions surrounding the underlying causes of myocarditis and inflammatory cardiomyopathy, as well as the available diagnosis and medical intervention. However, the emergence of innovative methodologies, novel therapeutic agents, and the application of cutting-edge computational modelling techniques along with a renewed focus on repurposed treatments, hold the promise of addressing these knowledge lacunae in the immediate years ahead. The employment of specific diagnostic strategies for varied clinical contexts is instrumental in enhancing the categorisation of individuals diagnosed with inflammatory cardiomyopathy, thereby fostering a more uniform nomenclature within a domain that is characterised by a certain degree of ambiguity. A pivotal aspect that demands attention pertains to the evaluation of the efficacy of numerous extant, repurposed, or emerging therapeutic interventions. This evaluation should be conducted through the framework of large-scale, controlled, and randomised clinical trials. Such an approach is deemed essential to pave the way for the formulation of aetiology-based therapeutic modalities [160,161,162].

## Figures and Tables

**Table 1:** Different types of immune cells involved in heart inflammation.

Proinflammatory and Immunomodulatory Cell Phenotype	How It Works	Findings
Monocytes [85,96,113,114,115,116]	Infiltrate sites of cardiac inflammation and damage in response to chemokine signals Murine models. Pro-inflammatory CD115^+^CD11b^+^Ly6C^high^CCR2^high^CX3CR1l^ow^ and CD115^+^CD11b^+^Ly6C^middle^CCR2^high^CX3CR1^low^ monocyte subsets Humans. The counterparts are represented by CD14^++^CD16−CCR2^high^CX3CR1^low^ and CD14^++^CD16^+^CCR2^middle^CX3CR1^high^CCR5^+^.	Monocytes become pro-inflammatory macrophages, which secrete TNF and IL-6, contributing to tissue degradation and T cell activation. CD115^+^CD11b^+^Ly6ClowCCR2^low^CX3CR1^high^ monocytes (human non-classic CD14^+^CD16^++^CCR2^low^CX3CR1^high^ monocytes) exhibit a propensity to be attracted to inflamed cardiac tissue. Monocytes can become macrophages which release anti-inflammatory cytokines and aid tissue repair. Cardiac fibroblasts release chemokines that induce monocyte migration to the myocardium that enable monocyte differentiation into macrophages in murine models of myocarditis.
Neutrophils [86,93,94,95,96,97,98,99,100,107,108,109,110,111,112]	Neutrophils can sustain inflammation via a process called NETosis, which involves the formation of neutrophil extracellular traps. NETosis has been observed to cause heart inflammation in murine models of experimental autoimmune myocarditis (EAM). The severity of heart inflammation is linked to the presence of neutrophils in the heart. The alarmins S100A8 and S100A9 promote inflammatory cardiomyopathy. S100A9−/− mice were resistant to the effects of CVB3 infection.	Neutrophils in the heart and the pancreas have been observed following CVB3 infection. The depletion of neutrophils in mice infected with CVB3 has been demonstrated to result in a reduction of myocarditis. Neutrophil count is linked to the level of cardiomyocyte death (measured by troponin I levels) in patients with acute coronary syndrome. S100A8–S100A9 levels are higher in patients with acute myocarditis than in healthy individuals.
Mast cells, natural killer cells and dendritic cells [102,103,104,105,106]	Mast cells release cytokines like TNF, IL-1β and IL-4 after infection. A high number of mast cells have been found in mice susceptible to autoimmune heart disease after CVB3 infection. Receptor NKG2D in natural killer cells protect against progression to inflammatory cardiomyopathy, leading to effective clearance of CVB3 from the heart. Dendritic cells migrate to regional lymph nodes and the spleen to present antigens to B and T cells, thereby activating the immune response.	Mouse studies have shown that dendritic cells are important antigen-presenting cells. Eliminating these cells can prevent heart failure. Dendritic cells present endogenously derived antigens triggering an autoimmune response in heart muscle tissue. Myosin peptide-loaded dendritic cells have created an experimental model for studying myosin-induced autoimmune myocarditis. Dendritic cells have been observed in mice’s hearts after infection with a virus. This happens at the same time as the build-up of certain immune cells.
T cells and B cells [81,82,83,84,85,86,87,88,89,90,91,92,93,94,95,96,117,118,119,120,121,122,123,124,125,126]	The T cell system has been identified as the primary instigator of both autoimmune myocarditis and inflammatory cardiomyopathy. The presence of activated T cells is an important factor in the development of heart damage in cases of viral myocarditis. CVB3-induced myocarditis revealed that disease severity differed in those lacking the CD8 or CD4 receptor, compared to their wild-type counterparts. Distinct T cell subsets have varied roles in virus-induced myocarditis (105). The inactivation of CD8+ T cells in mice resulted in exacerbated CVB3-induced cardiac injury and chronic myocarditis.	Mice lacking the T-box transcription factor TBX21 (T-bet) are prone to autoimmune myocarditis due to IL-17 production. In murine models of EAM, TH17 cells have been shown to promote DCM. Treg cells, reduced in patients with myocarditis or DCM, protect against CVB3-induced myocarditis in mice by reducing cardiac inflammation. IL-23 increases the ratio of TH17 cells to Treg cells by promoting the maturation of TH17 cells, which is important for autoimmune myocarditis in mice.
Eosinophils [131,132,133,134]	Patients with eosinophilia often develop heart problems. Eosinophils cause heart inflammation in response to parasites or drugs, and this can lead to heart failure. Hypereosinophilic mice with EAM exhibited a more rapid progression to DCM, a process mediated by eosinophil-derived IL-4. The eosinophil cationic protein plays a significant role in the pathogenesis of eosinophilic myocarditis in mice.	The major basic protein in eosinophilic granules is thrombogenic and contributes to vascular thromboembolism in patients with eosinophilic myocarditis. Eosinophil-deficient mice with EAM were found to be protected from developing DCM. Treatment with mepolizumab, an anti-IL-5 antibody, was effective in a patient with eosinophilic myocarditis.

Abbreviations: CVB3, coxsackievirus B3; DCM, dilated cardiomyopathy; EAM, experimental autoimmune myocarditis; IL, interleukin; NET, neutrophil extracellular traps; NK, natural killer; TBX21, T-box transcription factor; TH, T-helper; TNF, tumor necrosis factor.

## Data Availability

Not applicable.

## Supplementary Materials

The following supporting information can be downloaded at: https://www.mdpi.com/article/10.3390/v17040484/s1, Figure S1; A: Virulence; B: Treatment for associated heart disease; Figure S2: Prominent viruses associated with inflammatory cardiomyopathy over time; Table S1: Future priorities and research gaps to explore the viral function; Table S2: Future priorities and research gaps to explore the function of immune cells; Table S3: Technological Advancements, Clinical Applications and Cost Considerations; Table S4: Future priorities and research gaps for treating patients.

## References

[1] Richardson P., McKenna W., Bristow M., Maisch B., Mautner B., O’Connell J., Olsen E., Thiene G., Goodwin J., Gyarfas I. (1996). Report of the 1995 World Health Organization/International Society and Federation of Cardiology task force on the definition and classification of cardiomyopathies. Circulation.

[2] Giles T.D. (1997). New WHO/ISFC classification of cardiomyopathies: A task not completed. Circulation.

[3] Sánchez Torres R.J., Calderón R. (2004). The cardiomyopathies, a review for the primary physician. Puerto Rico Health Sci. J..

[4] Caforio A.L., Pankuweit S., Arbustini E., Basso C., Gimeno-Blanes J., Felix S.B., Fu M., Heliö T., Heymans S., Jahns R. (2013). Current state of knowledge on aetiology, diagnosis, management, and therapy of myocarditis: A position statement of the European Society of Cardiology Working Group on Myocardial and Pericardial Diseases. Eur. Heart J..

[5] Şahan E., Şahan S., Karamanlıoğlu M., Gul M., Tufekcioğlu O. (2016). The MOGE(S) classification: A TNM-like classification for cardiomyopathies. Herz.

[6] Ammirati E., Cipriani M., Moro C., Raineri C., Pini D., Sormani P., Mantovani R., Varrenti M., Pedrotti P., Conca C. (2018). Clinical presentation and outcome in a contemporary cohort of patients with acute myocarditis. Circulation.

[7] Ammirati E., Varrenti M., Sormani P., Bernasconi D., Moro C., Grosu A., D’Elia S., Raineri C., Quattrocchi G., Milazzo A. (2024). Long-term prognostic performance of cardiac magnetic resonance imaging markers versus complicated clinical presentation after an acute myocarditis. Int. J. Cardiol..

[8] Ginsberg F., Parrillo J.E. (2013). Fulminant myocarditis. Crit. Care Clin..

[9] Van Diepen S., Katz J.N., Albert N.M., Henry T.D., Jacobs A.K., Kapur N.K., Kilic A., Menon V., Ohman E.M., Sweitzer N.K. (2017). Contemporary Management of Cardiogenic Shock: A Scientific Statement from the American Heart Association. Circulation.

[10] Kociol R.D., Cooper L.T., Fang J.C., Moslehi J.J., Pang P.S., Sabe M.A., Shah R.V., Sims D.B., Thiene G., Vardeny O. (2020). Recognition and initial management of fulminant myocarditis: A scientific statement from the American Heart Association. Circulation.

[11] Chou H.W., Wang C.H., Lin L.Y., Chi N.H., Chou N.K., Yu H.Y., Chen Y.S. (2020). Prognostic factors for heart recovery in adult patients with acute fulminant myocarditis and cardiogenic shock supported with extracorporeal membrane oxygenation. J. Crit. Care.

[12] Ammirati E., Cipriani M., Lilliu M., Sormani P., Varrenti M., Raineri C., Petrella D., Garascia A., Pedrotti P., Roghi A. (2017). Survival and Left Ventricular Function Changes in Fulminant Versus Nonfulminant Acute Myocarditis. Circulation.

[13] Ammirati E., Veronese G., Brambatti M., Merlo M., Cipriani M., Potena L., Sormani P., Aoki T., Sugimura K., Sawamura A. (2019). Fulminant versus acute nonfulminant myocarditis in patients with left ventricular systolic dysfunction. J. Am. Coll. Cardiol..

[14] Dominguez F., Kuhl U., Pieske B., Garcia-Pavia P., Tschöpe C. (2016). Update on myocarditis and inflammatory cardiomyopathy: Reemergence of endomyocardial biopsy. Rev. Esp. Cardiol. (Engl. Ed)..

[15] Trachtenberg B.H., Hare J.M. (2017). Inflammatory cardiomyopathic syndromes. Circ. Res..

[16] Hu J.R., Florido R., Lipson E.J., Naidoo J., Ardehali R., Tocchetti C.G., Lyon A.R., Padera R.F., Johnson D.B., Moslehi J. (2019). Cardiovascular toxicities associated with immune checkpoint inhibitors. Cardiovasc. Res..

[17] Caforio A.L.P., Adler Y., Agostini C., Allanore Y., Anastasakis A., Arad M., Böhm M., Charron P., Elliott P.M., Eriksson U. (2017). Diagnosis and management of myocardial involvement in systemic immune-mediated diseases: A position statement of the European Society of Cardiology Working Group on Myocardial and Pericardial Disease. Eur. Heart J..

[18] Kuhl U., Pauschinger M., Noutsias M., Seeberg B., Bock T., Lassner D., Poller W., Kandolf R., Schultheiss H.P. (2005). High prevalence of viral genomes and multiple viral infections in the myocardium of adults with “idiopathic” left ventricular dysfunction. Circulation.

[19] Ukimura A., Satomi H., Ooi Y., Kanzaki Y. (2012). Myocarditis associated with influenza A H1N1pdm2009. Influenza Res. Treat..

[20] Van Linthout S., Klingel K., Tschope C. (2020). SARS-CoV2-related myocarditis-like syndroms: Shakespeare’s question: What’s in a name?. Eur. J. Heart Fail..

[21] Bozkurt B., Colvin M., Cook J., Cooper L.T., Deswal A., Fonarow G.C., Francis G.S., Lenihan D., Lewis E.F., McNamara D.M. (2016). Current diagnostic and treatment strategies for specific dilated cardiomyopathies: A scientific statement from the American Heart Association. Circulation.

[22] Pauschinger M., Bowles N.E., Fuentes-Garcia F.J., Pham V., Kühl U., Schwimmbeck P.L., Schultheiss H.P., Towbin J.A. (1999). Detection of adenoviral genome in the myocardium of adult patients with idiopathic left ventricular dysfunction. Circulation.

[23] Yen M.H., Huang Y.C., Chen M.C., Liu C.C., Chiu N.C., Lien R., Chang L.Y., Chiu C.H., Tsao K.C., Lin T.Y. (2015). Effect of intravenous immunoglobulin for neonates with severe enteroviral infections with emphasis on the timing of administration. J. Clin. Virol..

[24] Abzug M.J., Michaels M.G., Wald E., Jacobs R.F., Romero J.R., Sánchez P.J., Wilson G., Krogstad P., Storch G.A., Lawrence R. (2016). A randomized, double-blind, placebocontrolled trial of pleconaril for the treatment of neonates with enterovirus sepsis. J. Pediatr. Infect. Dis. Soc..

[25] Amdani S.M., Kim H.S., Orvedahl A., John A.O., Said A., Simpson K. (2018). Successful treatment of fulminant neonatal enteroviral myocarditis in monochorionic diamniotic twins with cardiopulmonary support, intravenous immunoglobulin and pocapavir. BMJ Case Rep..

[26] Schultz J.C., Hilliard A.A., Cooper L.T., Rihal C.S. (2009). Diagnosis and treatment of viral myocarditis. Mayo Clin. Proc..

[27] Woodruff J.F. (1980). Viral myocarditis. A review. Am. J. Pathol..

[28] Smith W.G. (1970). Coxsackie B myopericarditis in adults. Am. Heart J..

[29] Lyden D.C., Olszewski J., Feran M., Job L.P., Huber S.A. (1987). Coxsackievirus B-3-induced myocarditis. Effect of sex steroids on viremia and infectivity of cardiocytes. Am. J. Pathol..

[30] Zhou F., Yu T., Du R., Fan G., Liu Y., Liu Z., Xiang J., Wang Y., Song B., Gu X. (2020). Clinical course and risk factors for mortality of adult inpatients with COVID-19 in Wuhan, China: A retrospective cohort study. Lancet.

[31] Weiss P., Murdoch D.R. (2020). Clinical course and mortality risk of severe COVID-19. Lancet.

[32] Frisancho-Kiss S., Davis S.E., Nyland J.F., Frisancho J.A., Cihakova D., Barrett M.A., Rose N.R., Fairweather D. (2007). Cutting edge: Cross-regulation by TLR4 and T cell Ig mucin-3 determines sex differences in inflammatory heart disease. J. Immunol..

[33] Frisancho-Kiss S., Coronado M.J., Frisancho J.A., Lau V.M., Rose N.R., Klein S.L., Fairweather D. (2009). Gonadectomy of male BALB/c mice increases Tim-3(+) alternatively activated M2 macrophages, Tim-3(+) T cells, Th2 cells and Treg in the heart during acute coxsackievirus-induced myocarditis. Brain Behav. Immun..

[34] Coronado M.J., Bruno K.A., Blauwet L.A., Tschöpe C., Cunningham M.W., Pankuweit S., van Linthout S., Jeon E.S., McNamara D.M., Krejčí J. (2019). Elevated sera sST2 is associated with heart failure in men ≤50 years old with myocarditis. J. Am. Heart Assoc..

[35] Aimo A., Januzzi J.L., Bayes-Genis A., Vergaro G., Sciarrone P., Passino C., Emdin M. (2019). Clinical and Prognostic Significance of sST2 in Heart Failure: JACC Review Topic of the Week. J. Am. Coll. Cardiol..

[36] Zhang S.-F., Tuo J.-L., Huang X.-B., Zhu X., Zhang D.-M., Zhou K., Yuan L., Luo H.-J., Zheng B.-J., Yuen K.-Y. (2018). Epidemiology characteristics of human coronaviruses in patients with respiratory infection symptoms and phylogenetic analysis of HCoV-OC43 during 2010–2015 in Guangzhou. PLoS ONE.

[37] Cui J., Li F., Shi Z.L. (2019). Origin and evolution of pathogenic coronaviruses. Nat. Rev. Microbiol..

[38] Zhou P., Yang X.-L., Wang X.-G., Hu B., Zhang L., Zhang W., Si H.-R., Zhu Y., Li B., Huang C.-L. (2020). A pneumonia outbreak associated with a new coronavirus of probable bat origin. Nature.

[39] Wang D., Hu B., Hu C., Zhu F., Liu X., Zhang J., Wang B., Xiang H., Cheng Z., Xiong Y. (2020). Clinical characteristics of 138 hospitalized patients with 2019 novel coronavirus-infected pneumonia in Wuhan, China. JAMA.

[40] Zheng Y.Y., Ma Y.T., Zhang J.Y., Xie X. (2020). COVID-19 and the cardiovascular system. Nat. Rev. Cardiol..

[41] Nappi F., Nappi P., Gambardella I., Avtaar Singh S.S. (2022). Thromboembolic Disease and Cardiac Thrombotic Complication in COVID-19: A Systematic Review. Metabolites.

[42] Nappi F., Bellomo F., Avtaar Singh S.S. (2022). Insights into the Role of Neutrophils and Neutrophil Extracellular Traps in Causing Cardiovascular Complications in Patients with COVID-19: A Systematic Review. J. Clin. Med..

[43] Nappi F., Giacinto O., Ellouze O., Nenna A., Avtaar Singh S.S., Chello M., Bouzguenda A., Copie X. (2022). Association between COVID-19 Diagnosis and Coronary Artery Thrombosis: A Narrative Review. Biomedicines.

[44] Huang C., Wang Y., Li X., Ren L., Zhao J., Hu Y., Zhang L., Fan G., Xu J., Gu X. (2020). Clinical features of patients infected with 2019 novel coronavirus in Wuhan, China. Lancet.

[45] Srivastava A., Sundararaj S.N., Bhatia J., Arya D.S. (2024). Understanding long COVID myocarditis: A comprehensive review. Cytokine.

[46] Nappi F., Avtaar Singh S.S. (2023). SARS-CoV-2-Induced Myocarditis: A State-of-the-Art Review. Viruses.

[47] Guo J., Huang Z., Lin L., Lv J. (2020). Coronavirus disease 2019 (COVID-19) and cardiovascular disease: A viewpoint on the potential influence of angiotensin converting enzyme inhibitors/angiotensin receptor blockers on onset and severity of severe acute respiratory syndrome coronavirus 2 infection. J. Am. Heart Assoc..

[48] Santos R.A.S., Sampaio W.O., Alzamora A.C., Motta-Santos D., Alenina N., Bader M., Campagnole-Santos M.J. (2018). The ACE2/angiotensin-(1-7)/MAS axis of the renin-angiotensin system: Focus on angiotensin-(1-7). Physiol. Rev..

[49] Oudit G.Y., Kassiri Z., Jiang C., Liu P.P., Poutanen S.M., Penninger J.M., Butany J. (2009). SARS-coronavirus modulation of myocardial ACE2 expression and inflammation in patients with SARS. Eur. J. Clin. Investig..

[50] Li W. (2005). Receptor and viral determinants of SARS coronavirus adaptation to human ACE2. EMBO J..

[51] Hoffmann M., Kleine-Weber H., Schroeder S., Krüger N., Herrler T., Erichsen S., Schiergens T.S., Herrler G., Wu N.H., Nitsche A. (2020). SARS-CoV-2 cell entry depends on ACE2 and TMPRSS2 and is blocked by a clinically proven protease inhibitor. Cell.

[52] Glowacka I., Bertram S., Müller M.A., Allen P., Soilleux E., Pfefferle S., Steffen I., Tsegaye T.S., He Y., Gnirss K. (2011). Evidence that TMPRSS2 activates the severe acute respiratory syndrome coronavirus spike protein for membrane fusion and reduces viral control by the humoral immune response. J. Virol..

[53] Nicin L., Abplanalp W.T., Mellentin H., Kattih B., Tombor L., John D., Schmitto J.D., Heineke J., Emrich F., Arsalan M. (2020). Cell type-specific expression of the putative SARS-CoV-2 receptor ACE2 in human hearts. Eur. Heart J..

[54] Tschöpe C., Ammirati E., Bozkurt B., Caforio A.L.P., Cooper L.T., Felix S.B., Hare J.M., Heidecker B., Heymans S., Hübner N. (2021). Myocarditis and inflammatory cardiomyopathy: Current evidence and future directions. Nat. Rev. Cardiol..

[55] He Y., Chipman P.R., Howitt J., Bator C.M., Whitt M.A., Baker T.S., Kuhn R.J., Anderson C.W., Freimuth P., Rossmann M.G. (2001). Interaction of coxsackievirus B3 with the full length coxsackievirus-adenovirus receptor. Nat. Struct. Biol..

[56] Badorff C., Lee G.H., Lamphear B.J., Martone M.E., Campbell K.P., Rhoads R.E., Knowlton K.U. (1999). Enteroviral protease 2A cleaves dystrophin: Evidence of cytoskeletal disruption in an acquired cardiomyopathy. Nat. Med..

[57] Lassner D., Siegismund C.S., Kühl U., Rohde M., Stroux A., Escher F., Schultheiss H.P. (2018). CCR5del32 genotype in human enteroviral cardiomyopathy leads to spontaneous virus clearance improved outcome compared to wild type CCR5. J. Transl. Med..

[58] Kuhl U., Lassner D., von Schlippenbach J., Poller W., Schultheiss H.P. (2012). Interferon-beta improves survival in enterovirus-associated cardiomyopathy. J. Am. Coll. Cardiol..

[59] Leveque N., Garcia M., Bouin A., Nguyen J.H.C., Tran G.P., Andreoletti L., Semler B.L. (2017). Functional consequences of RNA 5′-terminal deletions on coxsackievirus B3 RNA replication and ribonucleoprotein complex formation. J. Virol..

[60] Bouin A., Gretteau P.A., Wehbe M., Renois F., N’Guyen Y., Lévêque N., Vu M.N., Tracy S., Chapman N.M., Bruneval P. (2019). Enterovirus persistence in cardiac cells of patients with idiopathic dilated cardiomyopathy is linked to 5′ terminal genomic RNA-deleted viral populations with viral-encoded proteinase activities. Circulation.

[61] Maisch B. (2019). Cardio-immunology of myocarditis: Focus on immune mechanisms and treatment options. Front. Cardiovasc. Med..

[62] Manaresi E., Gallinella G. (2019). Advances in the development of antiviral strategies against parvovirus B19. Viruses.

[63] Duechting A., Tschöpe C., Kaiser H., Lamkemeyer T., Tanaka N., Aberle S., Lang F., Torresi J., Kandolf R., Bock C.T. (2008). Human parvovirus B19 NS1 protein modulates inflammatory signaling by activation of STAT3/PIAS3 in human endothelial cells. J. Virol..

[64] Van Linthout S., Elsanhoury A., Klein O., Sosnowski M., Miteva K., Lassner D., Abou-El-Enein M., Pieske B., Kühl U., Tschöpe C. (2018). Telbivudine in chronic lymphocytic myocarditis and human parvovirus B19 transcriptional activity. ESC Heart Fail..

[65] Bultmann B.D., Sotlar K., Klingel K. (2004). Parvovirus B19. N. Engl. J. Med..

[66] Kindermann I., Kindermann M., Kandolf R., Klingel K., Bültmann B., Müller T., Lindinger A., Böhm M. (2008). Predictors of outcome in patients with suspected myocarditis. Circulation.

[67] Hjalmarsson C., Liljeqvist J.Å., Lindh M., Karason K., Bollano E., Oldfors A., Andersson B. (2019). Parvovirus B19 in endomyocardial biopsy of patients with idiopathic dilated cardiomyopathy: Foe or bystander?. J. Card. Fail..

[68] Schenk T., Enders M., Pollak S., Hahn R., Huzly D. (2009). High prevalence of human parvovirus B19 DNA in myocardial autopsy samples from subjects without myocarditis or dilative cardiomyopathy. J. Clin. Microbiol..

[69] Lotze U., Egerer R., Glück B., Zell R., Sigusch H., Erhardt C., Heim A., Kandolf R., Bock T., Wutzler P. (2010). Low level myocardial parvovirus B19 persistence is a frequent finding in patients with heart disease but unrelated to ongoing myocardial injury. J. Med. Virol..

[70] Koepsell S.A., Anderson D.R., Radio S.J. (2012). Parvovirus B19 is a bystander in adult myocarditis. Cardiovasc. Pathol..

[71] Bock C.T., Klingel K., Kandolf R. (2010). Human parvovirus B19-associated myocarditis. N. Engl. J. Med..

[72] Bock C.T., Düchting A., Utta F., Brunner E., Sy B.T., Klingel K., Lang F., Gawaz M., Felix S.B., Kandolf R. (2014). Molecular phenotypes of human parvovirus B19 in patients with myocarditis. World J. Cardiol..

[73] Dennert R., van Paassen P., Wolffs P., Bruggeman C., Velthuis S., Felix S., van Suylen R.J., Crijns H.J., Cohen Tervaert J.W., Heymans S. (2012). Differences in virus prevalence and load in the hearts of patients with idiopathic dilated cardiomyopathy with and without immune-mediated inflammatory diseases. Clin. Vaccine Immunol..

[74] Kuhl U., Lassner D., Dorner A., Rohde M., Escher F., Seeberg B., Hertel E., Tschope C., Skurk C., Gross U.M. (2013). A distinct subgroup of cardiomyopathy patients characterized by transcriptionally active cardiotropic erythrovirus and altered cardiac gene expression. Basic Res. Cardiol..

[75] Adamson-Small L.A., Ignatovich I.V., Laemmerhirt M.G., Hobbs J.A. (2014). Persistent parvovirus B19 infection in non-erythroid tissues: Possible role in the inflammatory and disease process. Virus Res..

[76] Richter J., Quintanilla-Martinez L., Bienemann K., Zeus T., Germing U., Sander O., Kandolf R., Häussinger D., Klingel K. (2013). An unusual presentation of a common infection. Infection.

[77] Kaufer B.B., Flamand L. (2014). Chromosomally integrated HHV-6: Impact on virus, cell and organismal biology. Curr. Opin. Virol..

[78] Barbaro G. (2005). HIV-associated cardiomyopathy etiopathogenesis and clinical aspects. Herz.

[79] Sanchez M.J., Bergasa N.V. (2008). Hepatitis C associated cardiomyopathy: Potential pathogenic mechanisms and clinical implications. Med. Sci. Monit..

[80] Kumar K., Guirgis M., Zieroth S., Lo E., Menkis A.H., Arora R.C., Freed D.H. (2011). Influenza myocarditis and myositis: Case presentation and review of the literature. Can. J. Cardiol..

[81] Swirski F.K., Nahrendorf M. (2018). Cardioimmunology: The immune system in cardiac homeostasis and disease. Nat. Rev. Immunol..

[82] Pollack A., Kontorovich A.R., Fuster V., Dec G.W. (2015). Viral myocarditis—Diagnosis, treatment options, and current controversies. Nat. Rev. Cardiol..

[83] Alexopoulou L., Holt A.C., Medzhitov R., Flavell R.A. (2001). Recognition of double-stranded RNA and activation of NF-kappaB by Toll-like receptor 3. Nature.

[84] Tschöpe C., Müller I., Xia Y., Savvatis K., Pappritz K., Pinkert S., Lassner D., Heimesaat M.M., Spillmann F., Miteva K. (2017). NOD2 (Nucleotide-Binding Oligomerization Domain 2) Is a Major Pathogenic Mediator of Coxsackievirus B3-Induced Myocarditis. Circ. Heart Fail..

[85] Miteva K., Pappritz K., Sosnowski M., El-Shafeey M., Müller I., Dong F., Savvatis K., Ringe J., Tschöpe C., Van Linthout S. (2018). Mesenchymal stromal cells inhibit NLRP3 inflammasome activation in a model of Coxsackievirus B3-induced inflammatory cardiomyopathy. Sci. Rep..

[86] Müller I., Vogl T., Kühl U., Krannich A., Banks A., Trippel T., Noutsias M., Maisel A.S., van Linthout S., Tschöpe C. (2020). Serum alarmin S100A8/S100A9 levels and its potential role as biomarker in myocarditis. ESC Heart Fail..

[87] Heymans S., Eriksson U., Lehtonen J., Cooper L.T. (2016). The Quest for New Approaches in Myocarditis and Inflammatory Cardiomyopathy. J. Am. Coll. Cardiol..

[88] Huang C.H., Vallejo J.G., Kollias G., Mann D.L. (2009). Role of the innate immune system in acute viral myocarditis. Basic Res. Cardiol..

[89] Libby P., Nahrendorf M., Swirski F.K. (2016). Leukocytes Link Local and Systemic Inflammation in Ischemic Cardiovascular Disease: An Expanded “Cardiovascular Continuum”. J. Am. Coll. Cardiol..

[90] Leuschner F., Rauch P.J., Ueno T., Gorbatov R., Marinelli B., Lee W.W., Dutta P., Wei Y., Robbins C., Iwamoto Y. (2012). Rapid monocyte kinetics in acute myocardial infarction are sustained by extramedullary monocytopoiesis. J. Exp. Med..

[91] Swirski F.K., Nahrendorf M., Etzrodt M., Wildgruber M., Cortez-Retamozo V., Panizzi P., Figueiredo J.L., Kohler R.H., Chudnovskiy A., Waterman P. (2009). Identification of splenic reservoir monocytes and their deployment to inflammatory sites. Science.

[92] Ismahil M.A., Hamid T., Bansal S.S., Patel B., Kingery J.R., Prabhu S.D. (2014). Remodeling of the mononuclear phagocyte network underlies chronic inflammation and disease progression in heart failure: Critical importance of the cardiosplenic axis. Circ. Res..

[93] Leuschner F., Courties G., Dutta P., Mortensen L.J., Gorbatov R., Sena B., Novobrantseva T.I., Borodovsky A., Fitzgerald K., Koteliansky V. (2015). Silencing of CCR2 in myocarditis. Eur. Heart J..

[94] Miteva K., Pappritz K., El-Shafeey M., Dong F., Ringe J., Tschöpe C., Van Linthout S. (2017). Mesenchymal Stromal Cells Modulate Monocytes Trafficking in Coxsackievirus B3-Induced Myocarditis. Stem Cells Transl. Med..

[95] Cooper L.T., Fairweather D. (2015). Nano-scale treatment for a macro-scale disease: Nanoparticle-delivered siRNA silences CCR2 and treats myocarditis. Eur. Heart J..

[96] Pappritz K., Savvatis K., Miteva K., Kerim B., Dong F., Fechner H., Müller I., Brandt C., Lopez B., González A. (2018). Immunomodulation by adoptive regulatory T-cell transfer improves Coxsackievirus B3-induced myocarditis. FASEB J..

[97] Müller I., Pappritz K., Savvatis K., Puhl K., Dong F., El-Shafeey M., Hamdani N., Hamman I., Noutsias M., Infante-Duarte C. (2017). CX3CR1 knockout aggravates Coxsackievirus B3-induced myocarditis. PLoS ONE.

[98] Klingel K., Stephan S., Sauter M., Zell R., McManus B.M., Bültmann B., Kandolf R. (1996). Pathogenesis of murine enterovirus myocarditis: Virus dissemination and immune cell targets. J. Virol..

[99] Hofmann P., Schmidtke M., Stelzner A., Gemsa D. (2001). Suppression of proinflammatory cytokines and induction of IL-10 in human monocytes after Coxsackievirus B3 infection. J. Med. Virol..

[100] Kandolf R., Sauter M., Aepinus C., Schnorr J.J., Selinka H.C., Klingel K. (1999). Mechanisms and consequences of enterovirus persistence in cardiac myocytes and cells of the immune system. Virus Res..

[101] Savvatis K., van Linthout S., Miteva K., Pappritz K., Westermann D., Schefold J.C., Fusch G., Weithäuser A., Rauch U., Becher P.-M. (2012). Mesenchymal stromal cells but not cardiac fibroblasts exert beneficial systemic immunomodulatory effects in experimental myocarditis. PLoS ONE.

[102] Fairweather D., Frisancho-Kiss S., Gatewood S., Njoku D., Steele R., Barrett M., Rose N.R. (2004). Mast cells and innate cytokines are associated with susceptibility to autoimmune heart disease following coxsackievirus B3 infection. Autoimmunity.

[103] Klingel K., Fabritius C., Sauter M., Göldner K., Stauch D., Kandolf R., Ettischer N., Gahlen S., Schönberger T., Ebner S. (2014). The activating receptor NKG2D of natural killer cells promotes resistance against enterovirus-mediated inflammatory cardiomyopathy. J. Pathol..

[104] Yuan J., Liu Z., Lim T., Zhang H., He J., Walker E., Shier C., Wang Y., Su Y., Sall A. (2009). CXCL10 inhibits viral replication through recruitment of natural killer cells in coxsackievirus B3-induced myocarditis. Circ. Res..

[105] Clemente-Casares X., Hosseinzadeh S., Barbu I., Dick S.A., Macklin J.A., Wang Y., Momen A., Kantores C., Aronoff L., Farno M. (2017). A CD103(+) conventional dendritic cell surveillance system prevents development of overt heart failure during subclinical viral myocarditis. Immunity.

[106] Eriksson U., Ricci R., Hunziker L., Kurrer M.O., Oudit G.Y., Watts T.H., Sonderegger I., Bachmaier K., Kopf M., Penninger J.M. (2003). Dendritic cell-induced autoimmune heart failure requires cooperation between adaptive and innate immunity. Nat. Med..

[107] Xu D., Wang P., Yang J., Qian Q., Li M., Wei L., Xu W. (2018). Gr-1+ cells other than Ly6G+ neutrophils limit virus replication and promote myocardial inflammation and fibrosis following coxsackievirus B3 infection of mice. Front. Cell Infect. Microbiol..

[108] Rivadeneyra L., Charó N., Kviatcovsky D., de la Barrera S., Gómez R.M., Schattner M. (2018). Role of neutrophils in CVB3 infection and viral myocarditis. J. Mol. Cell Cardiol..

[109] Weckbach L.T., Grabmaier U., Uhl A., Gess S., Boehm F., Zehrer A., Pick R., Salvermoser M., Czermak T., Pircher J. (2019). Midkine drives cardiac inflammation by promoting neutrophil trafficking and NETosis in myocarditis. J. Exp. Med..

[110] Afanasyeva M., Georgakopoulos D., Belardi D.F., Ramsundar A.C., Barin J.G., Kass D.A., Rose N.R. (2004). Quantitative analysis of myocardial inflammation by flow cytometry in murine autoimmune myocarditis: Correlation with cardiac function. Am. J. Pathol..

[111] Müller I., Vogl T., Pappritz K., Miteva K., Savvatis K., Rohde D., Most P., Lassner D., Pieske B., Kühl U. (2017). Pathogenic role of the damageassociated molecular patterns S100A8 and S100A9 in coxsackievirus B3-induced myocarditis. Circ. Heart Fail..

[112] Tahto E., Jadric R., Pojskic L., Kicic E. (2017). Neutrophilto-lymphocyte ratio and its relation with markers of inflammation and myocardial necrosis in patients with acute coronary syndrome. Med. Arch..

[113] Nahrendorf M., Swirski F.K. (2013). Monocyte and macrophage heterogeneity in the heart. Circ. Res..

[114] Yang J., Zhang L., Yu C., Yang X.F., Wang H. (2014). Monocyte and macrophage differentiation: Circulation inflammatory monocyte as biomarker for inflammatory diseases. Biomark. Res..

[115] Pappritz K., Savvatis K., Koschel A., Miteva K., Tschöpe C., Van Linthout S. (2018). Cardiac (myo)fibroblasts modulate the migration of monocyte subsets. Sci. Rep..

[116] Hou X., Chen G., Bracamonte-Baran W., Choi H.S., Diny N.L., Sung J., Hughes D., Won T., Wood M.K., Talor M.V. (2019). The cardiac microenvironment instructs divergent monocyte fates and functions in myocarditis. Cell Rep..

[117] Liu P., Aitken K., Kong Y.Y., Opavsky M.A., Martino T., Dawood F., Wen W.H., Kozieradzki I., Bachmaier K., Straus D. (2000). The tyrosine kinase p56lck is essential in coxsackievirus B3-mediated heart disease. Nat. Med..

[118] Baldeviano G.C., Barin J.G., Talor M.V., Srinivasan S., Bedja D., Zheng D., Gabrielson K., Iwakura Y., Rose N.R., Cihakova D. (2010). Interleukin-17A is dispensable for myocarditis but essential for the progression to dilated cardiomyopathy. Circ. Res..

[119] Shi Y., Fukuoka M., Li G., Liu Y., Chen M., Konviser M., Chen X., Opavsky M.A., Liu P.P. (2010). Regulatory T cells protect mice against coxsackievirus-induced myocarditis through the transforming growth factor β-coxsackie-adenovirus receptor pathway. Circulation.

[120] Anzai A., Mindur J.E., Halle L., Sano S., Choi J.L., He S., McAlpine C.S., Chan C.T., Kahles F., Valet C. (2019). Self-reactive CD4(+) IL-3(+) T cells amplify autoimmune inflammation in myocarditis by inciting monocyte chemotaxis. J. Exp. Med..

[121] Opavsky M.A., Penninger J., Aitken K., Wen W.H., Dawood F., Mak T., Liu P. (1999). Susceptibility to myocarditis is dependent on the response of alphabeta T lymphocytes to coxsackieviral infection. Circ. Res..

[122] Klingel K., Schnorr J.J., Sauter M., Szalay G., Kandolf R. (2003). β2-Microglobulin-associated regulation of interferon-γ and virus-specific immunoglobulin G confer resistance against the development of chronic coxsackievirus myocarditis. Am. J. Pathol..

[123] Rangachari M., Mauermann N., Marty R.R., Dirnhofer S., Kurrer M.O., Komnenovic V., Penninger J.M., Eriksson U. (2006). T-bet negatively regulates autoimmune myocarditis by suppressing local production of interleukin 17. J. Exp. Med..

[124] Myers J.M., Cooper L.T., Kem D.C., Stavrakis S., Kosanke S.D., Shevach E.M., Fairweather D., Stoner J.A., Cox C.J., Cunningham M.W. (2016). Cardiac myosin-Th17 responses promote heart failure in human myocarditis. JCI Insight.

[125] Ahern P.P., Schiering C., Buonocore S., McGeachy M.J., Cua D.J., Maloy K.J., Powrie F. (2010). Interleukin-23 drives intestinal inflammation through direct activity on T cells. Immunity.

[126] Wu L., Diny N.L., Ong S., Barin J.G., Hou X., Rose N.R., Talor M.V., Čiháková D. (2016). Pathogenic IL-23 signaling is required to initiate GM-CSF-driven autoimmune myocarditis in mice. Eur. J. Immunol..

[127] Kaya Z., Leib C., Katus H.A. (2012). Autoantibodies in heart failure and cardiac dysfunction. Circ. Res..

[128] Weber M.S., Prod’homme T., Patarroyo J.C., Molnarfi N., Karnezis T., Lehmann-Horn K., Danilenko D.M., Eastham-Anderson J., Slavin A.J., Linington C. (2010). B-cell activation influences T-cell polarization and outcome of anti-CD20 B-cell depletion in central nervous system autoimmunity. Ann. Neurol..

[129] Zouggari Y., Ait-Oufella H., Bonnin P., Simon T., Sage A.P., Guérin C., Vilar J., Caligiuri G., Tsiantoulas D., Laurans L. (2013). B lymphocytes trigger monocyte mobilization and impair heart function after acute myocardial infarction. Nat. Med..

[130] Tschope C., Van Linthout S., Spillmann F., Posch M.G., Reinke P., Volk H.D., Elsanhoury A., Kühl U. (2019). Targeting CD20+ B-lymphocytes in inflammatory dilated cardiomyopathy with rituximab improves clinical course: A case series. Eur. Heart J. Case Rep..

[131] Diny N., Baldeviano G.C.L., Talor M.V., Barin J.G., Ong S., Bedja D., Hays A.G., Gilotra N.A., Coppens I., Rose N.R. (2017). Eosinophil-derived IL-4 drives progression of myocarditis to inflammatory dilated cardiomyopathy. J. Exp. Med..

[132] Tai P.C., Ackerman S.J., Spry C.J., Dunnette S., Olsen E.G., Gleich G.J. (1987). Deposits of eosinophil granule proteins in cardiac tissues of patients with eosinophilic endomyocardial disease. Lancet.

[133] Thambidorai S.K., Korlakunta H.L., Arouni A.J., Hunter W.J., Holmberg M.J. (2009). Acute eosinophilic myocarditis mimicking myocardial infarction. Tex. Heart Inst. J..

[134] Song T., Jones D.M., Homsi Y. (2017). Therapeutic effect of anti-IL-5 on eosinophilic myocarditis with large pericardial effusion. BMJ Case Rep..

[135] Mahon N.G., Madden B.P., Caforio A.L., Elliott P.M., Haven A.J., Keogh B.E., Davies M.J., McKenna W.J. (2002). Immunohistologic evidence of myocardial disease in apparently healthy relatives of patients with dilated cardiomyopathy. J. Am. Coll. Cardiol..

[136] Caforio A.L., Keeling P.J., Zachara E., Mestroni L., Camerini F., Mann J.M., Bottazzo G.F., McKenna W.J. (1994). Evidence from family studies for autoimmunity in dilated cardiomyopathy. Lancet.

[137] Caforio A.L., Mahon N.G., Baig M.K., Tona F., Murphy R.T., Elliott P.M., McKenna W.J. (2007). Prospective familial assessment in dilated cardiomyopathy: Cardiac autoantibodies predict disease development in asymptomatic relatives. Circulation.

[138] Mestroni L., Rocco C., Gregori D., Sinagra G., Di Lenarda A., Miocic S., Vatta M., Pinamonti B., Muntoni F., Caforio A.L. (1999). Familial dilated cardiomyopathy: Evidence for genetic and phenotypic heterogeneity. J. Am. Coll. Cardiol..

[139] Neu N., Rose N.R., Beisel K.W., Herskowitz A., Gurri-Glass G., Craig S.W. (1987). Cardiac myosin induces myocarditis in genetically predisposed mice. J. Immunol..

[140] Smith S.C., Allen P.M. (1991). Myosin-induced acute myocarditis is a T cell-mediated disease. J. Immunol..

[141] Li Y., Heuser J.S., Cunningham L.C., Kosanke S.D., Cunningham M.W. (2006). Mimicry and antibody-mediated cell signaling in autoimmune myocarditis. J. Immunol..

[142] Frustaci A., Chimenti C., Calabrese F., Pieroni M., Thiene G., Maseri A. (2003). Immunosuppressive therapy for active lymphocytic myocarditis: Virological and immunologic profile of responders versus nonresponders. Circulation.

[143] Frustaci A., Russo M.A., Chimenti C. (2009). Randomized study on the efficacy of immunosuppressive therapy in patients with virus-negative inflammatory cardiomyopathy: The TIMIC study. Eur. Heart J..

[144] Escher F., Kühl U., Lassner D., Poller W., Westermann D., Pieske B., Tschöpe C., Schultheiss H.P. (2016). Long-term outcome of patients with virus-negative chronic myocarditis or inflammatory cardiomyopathy after immunosuppressive therapy. Clin. Res. Cardiol..

[145] Caforio A.L., Bonifacio E., Stewart J.T., Neglia D., Parodi O., Bottazzo G.F., McKenna W.J. (1990). Novel organ-specific circulating cardiac autoantibodies in dilated cardiomyopathy. J. Am. Coll. Cardiol..

[146] Caforio A.L., Calabrese F., Angelini A., Tona F., Vinci A., Bottaro S., Ramondo A., Carturan E., Iliceto S., Thiene G. (2007). A prospective study of biopsy-proven myocarditis: Prognostic relevance of clinical and aetiopathogenetic features at diagnosis. Eur. Heart J..

[147] Caforio A.L., Grazzini M., Mann J.M., Keeling P.J., Bottazzo G.F., McKenna W.J., Schiaffino S. (1992). Identification of alpha-and beta-cardiac myosin heavy chain isoforms as major autoantigens in dilated cardiomyopathy. Circulation.

[148] Schulze K., Becker B.F., Schultheiss H.P. (1989). Antibodies to the ADP/ATP carrier, an autoantigen in myocarditis and dilated cardiomyopathy, penetrate into myocardial cells and disturb energy metabolism in vivo. Circ. Res..

[149] Caforio A.L., Angelini A., Blank M., Shani A., Kivity S., Goddard G., Doria A., Schiavo A., Testolina M., Bottaro S. (2015). Passive transfer of affinity-purified anti-heart autoantibodies (AHA) from sera of patients with myocarditis induces experimental myocarditis in mice. Int. J. Cardiol..

[150] Zwacka R.M., Zhou W., Zhang Y., Darby C.J., Dudus L., Halldorson J., Oberley L., Engelhardt J.F. (1998). Redox gene therapy for ischemia/reperfusion injury of the liver reduces AP1 and NF-kappaB activation. Nat. Med..

[151] Jahns R., Boivin V., Hein L., Triebel S., Angermann C.E., Ertl G., Lohse M.J. (2004). Direct evidence for a beta 1-adrenergic receptor-directed autoimmune attack as a cause of idiopathic dilated cardiomyopathy. J. Clin. Invest..

[152] Nishimura H., Okazaki T., Tanaka Y., Nakatani K., Hara M., Matsumori A., Sasayama S., Mizoguchi A., Hiai H., Minato N. (2001). Autoimmune dilated cardiomyopathy in PD-1 receptor-deficient mice. Science.

[153] Meder B., Rühle F., Weis T., Homuth G., Keller A., Franke J., Peil B., Lorenzo Bermejo J., Frese K., Huge A. (2014). A genome-wide association study identifies 6p21 as novel risk locus for dilated cardiomyopathy. Eur. Heart J..

[154] Arbustini E., Narula N., Dec G.W., Reddy K.S., Greenberg B., Kushwaha S., Marwick T., Pinney S., Bellazzi R., Favalli V. (2013). The MOGE(S) classification for a phenotype-genotype nomenclature of cardiomyopathy: Endorsed by the World Heart Federation. J. Am. Coll. Cardiol..

[155] Hazebroek M.R., Moors S., Dennert R., van den Wijngaard A., Krapels I., Hoos M., Verdonschot J., Merken J.J., de Vries B., Wolffs P.F. (2015). Prognostic Relevance of Gene-Environment Interactions in Patients with Dilated Cardiomyopathy: Applying the MOGE(S) Classification. J. Am. Coll. Cardiol..

[156] Pinto Y.M., Elliott P.M., Arbustini E., Adler Y., Anastasakis A., Böhm M., Duboc D., Gimeno J., de Groote P., Imazio M. (2016). Proposal for a revised definition of dilated cardiomyopathy, hypokinetic non-dilated cardiomyopathy, and its implications for clinical practice: A position statement of the ESC working group on myocardial and pericardial diseases. Eur. Heart J..

[157] Gil-Cruz C., Perez-Shibayama C., De Martin A., Ronchi F., van der Borght K., Niederer R., Onder L., Lütge M., Novkovic M., Nindl V. (2019). Microbiota-derived peptide mimics drive lethal inflammatory cardiomyopathy. Science.

[158] Corrado D., Basso C., Thiene G. (2001). Sudden cardiac death in young people with apparently normal heart. Cardiovasc. Res..

[159] Aquaro G.D., Perfetti M., Camastra G., Monti L., Dellegrottaglie S., Moro C., Pepe A., Todiere G., Lanzillo C., Scatteia A. (2017). Cardiac MR with late gadolinium enhancement in acute myocarditis with preserved systolic function: ITAMY study. J. Am. Coll. Cardiol..

[160] Kasner M., Aleksandrov A., Escher F., Al-Saadi N., Makowski M., Spillmann F., Genger M., Schultheiss H.P., Kühl U., Pieske B. (2017). Multimodality imaging approach in the diagnosis of chronic myocarditis with preserved left ventricular ejection fraction (MCpEF): The role of 2D speckle-tracking echocardiography. Int. J. Cardiol..

[161] Ammirati E., Veronese G., Cipriani M., Moroni F., Garascia A., Brambatti M., Adler E.D., Frigerio M. (2018). Acute and fulminant myocarditis: A pragmatic clinical approach to diagnosis and 61treatment. Curr. Cardiol. Rep..

[162] Tschope C., Cooper L.T., Torre-Amione G., Van Linthout S. (2019). Management of myocarditis-related cardiomyopathy in adults. Circ. Res..

[163] Merlo M., Ammirati E., Gentile P., Artico J., Cannatà A., Finocchiaro G., Barbati G., Sormani P., Varrenti M., Perkan A. (2019). Persistent left ventricular dysfunction after acute lymphocytic myocarditis: Frequency and predictors. PLoS ONE.

[164] McMurray J.J., Adamopoulos S., Anker S.D., Auricchio A., Böhm M., Dickstein K., Falk V., Filippatos G., Fonseca C., Gomez-Sanchez M.A. (2012). ESC Guidelines for the diagnosis and treatment of acute and chronic heart failure 2012: The Task Force for the Diagnosis and Treatment of Acute and Chronic Heart Failure 2012 of the European Society of Cardiology. Developed in collaboration with the Heart Failure Association (HFA) of the ESC. Eur. Heart J..

[165] Ferreira V.M., Schulz-Menger J., Holmvang G., Kramer C.M., Carbone I., Sechtem U., Kindermann I., Gutberlet M., Cooper L.T., Liu P. (2018). Cardiovascular magnetic resonance in nonischemic myocardial inflammation: Expert recommendations. J. Am. Coll. Cardiol..

[166] Luetkens J.A., Faron A., Isaak A., Dabir D., Kuetting D., Feisst A., Schmeel F.C., Sprinkart A.M., Thomas D. (2019). Comparison of original and 2018 Lake Louise criteria for diagnosis of acute myocarditis: Results of a validation cohort. Radiol. Cardiothorac. Imaging.

[167] Thavendiranathan P., Walls M., Giri S., Verhaert D., Rajagopalan S., Moore S., Simonetti O.P., Raman S.V. (2012). Improved detection of myocardial involvement in acute inflammatory cardiomyopathies using T2 mapping. Circ. Cardiovasc. Imaging.

[168] Messroghli D.R., Moon J.C., Ferreira V.M., Grosse-Wortmann L., He T., Kellman P., Mascherbauer J., Nezafat R., Salerno M., Schelbert E.B. (2017). Clinical recommendations for cardiovascular magnetic resonance mapping of T1, T2, T2* and extracellular volume: A consensus statement by the Society for Cardiovascular Magnetic Resonance (SCMR) endorsed by the European Association for Cardiovascular Imaging (EACVI). J. Cardiovasc. Magn. Reson..

[169] Bohnen S., Radunski U.K., Lund G.K., Kandolf R., Stehning C., Schnackenburg B., Adam G., Blankenberg S., Muellerleile K. (2015). Performance of T1 and T2 mapping cardiovascular magnetic resonance to detect active myocarditis in patients with recent-onset heart failure. Circ. Cardiovasc. Imaging.

[170] Radunski U.K., Lund G.K., Säring D., Bohnen S., Stehning C., Schnackenburg B., Avanesov M., Tahir E., Adam G., Blankenberg S. (2017). T1 and T2 mapping cardiovascular magnetic resonance imaging techniques reveal unapparent myocardial injury in patients with myocarditis. Clin. Res. Cardiol..

[171] Lurz P., Luecke C., Eitel I., Föhrenbach F., Frank C., Grothoff M., de Waha S., Rommel K.P., Lurz J.A., Klingel K. (2016). Comprehensive cardiac magnetic resonance imaging in patients with suspected myocarditis: The MyoRacer-trial. J. Am. Coll. Cardiol..

[172] Puntmann V.O., Zeiher A.M., Nagel E. (2018). T1 and T2 mapping in myocarditis: Seeing beyond the horizon ofLake Louise criteria and histopathology. Expert Rev. Cardiovasc. Ther..

[173] Francone M., Chimenti C., Galea N., Scopelliti F., Verardo R., Galea R., Carbone I., Catalano C., Fedele F., Frustaci A. (2014). CMR sensitivity varies with clinical presentation and extent of cell necrosis in biopsyproven acute myocarditis. JACC Cardiovasc. Imaging.

[174] Tanacli R., Hashemi D., Lapinskas T., Edelmann F., Gebker R., Pedrizzetti G., Schuster A., Nagel E., Pieske B., Düngen H.D. (2019). Range variability in CMR feature tracking multilayer strain across different stages of heart failure. Sci. Rep..

[175] Escher F., Westermann D., Gaub R., Pronk J., Bock T., Al-Saadi N., Kühl U., Schultheiss H.P., Tschöpe C. (2011). Development of diastolic heart failure in a 6-year follow-up study in patients after acute myocarditis. Heart.

[176] Bohnen S., Radunski U.K., Lund G.K., Ojeda F., Looft Y., Senel M., Radziwolek L., Avanesov M., Tahir E., Stehning C. (2017). Tissue characterization by T1 and T2 mapping cardiovascular magnetic resonance imaging to monitor myocardial inflammation in healing myocarditis. Eur. Heart J. Cardiovasc. Imaging.

[177] Heidecker B., Ruedi G., Baltensperger N., Gresser E., Kottwitz J., Berg J., Manka R., Landmesser U., Lüscher T.F., Patriki D. (2019). Systematic use of cardiac magnetic resonance imaging in MINOCA led to a five-fold increase in the detection rate of myocarditis: A retrospective study. Swiss Med. Wkly..

[178] Patriki D., Gresser E., Manka R., Emmert M.Y., Lüscher T.F., Heidecker B. (2018). Approximation of the incidence of myocarditis by systematic screening with cardiac magnetic resonance imaging. JACC Heart Fail..

[179] Jessup M., Lindenfeld J. (2018). Light at the end of the myocarditis tunnel. JACC Heart Fail..

[180] Schneider J.E., Stojanovic I. (2019). Economic evaluation of cardiac magnetic resonance with fast-SENC in the diagnosis and management of early heart failure. Health Econ. Rev..

[181] Ge Y., Pandya A., Steel K., Bingham S., Jerosch-Herold M., Chen Y.Y., Mikolich J.R., Arai A.E., Bandettini W.P., Patel A.R. (2020). Cost-effectiveness analysis of stress cardiovascular magnetic resonance imaging for stable chest pain syndromes. JACC Cardiovasc. Imaging.

[182] Petrov G., Kelle S., Fleck E., Wellnhofer E. (2015). Incremental cost-effectiveness of dobutamine stress cardiac magnetic resonance imaging in patients at intermediate risk for coronary artery disease. Clin. Res. Cardiol..

[183] Cooper L.T., Baughman K.L., Feldman A.M., Frustaci A., Jessup M., Kuhl U., Levine G.N., Narula J., Starling R.C., Towbin J. (2007). The role of endomyocardial biopsy in the management of cardiovascular disease: A scientific statement from the American Heart Association, the American College of Cardiology, and the European Society of Cardiology. J. Am. Coll. Cardiol..

[184] Backhaus S.J., Lange T., Beuthner B.E., Topci R., Wang X., Kowallick J.T., Lotz J., Seidler T., Toischer K., Zeisberg E.M. (2020). Real-time cardiovascular magnetic resonance T1 and extracellular volume fraction mapping for tissue characterisation in aortic stenosis. Cardiovasc. Magn. Reson..

[185] Zhang S., Joseph A.A., Voit D., Schaetz S., Merboldt K.D., Unterberg-Buchwald C., Hennemuth A., Lotz J., Frahm J. (2014). Real-time magnetic resonance imaging of cardiac function and flow—Recent progress. Quant. Imaging Med. Surg..

[186] Lurz P., Eitel I., Adam J., Steiner J., Grothoff M., Desch S., Fuernau G., de Waha S., Sareban M., Luecke C. (2012). Diagnostic performance of CMR imaging compared with EMB in patients with suspected myocarditis. JACC Cardiovasc. Imaging.

[187] Gräni C., Eichhorn C., Bière L., Murthy V.L., Agarwal V., Kaneko K., Cuddy S., Aghayev A., Steigner M., Blankstein R. (2017). Prognostic value of cardiac magnetic resonance tissue characterization in risk stratifying patients with suspected myocarditis. J. Am. Coll. Cardiol..

[188] Schelbert E.B., Piehler K.M., Zareba K.M., Moon J.C., Ugander M., Messroghli D.R., Valeti U.S., Chang C.C., Shroff S.G., Diez J. (2015). Myocardial fibrosis quantified by extracellular volume is associated with subsequent hospitalization for heart failure, death, or both across the spectrum of ejection fraction and heart failure stage. J. Am. Heart Assoc..

[189] Mewton N., Liu C.Y., Croisille P., Bluemke D., Lima J.A. (2011). Assessment of myocardial fibrosis with cardiovascular magnetic resonance. J. Am. Coll. Cardiol..

[190] Berg J., Kottwitz J., Baltensperger N., Kissel C.K., Lovrinovic M., Mehra T., Scherff F., Schmied C., Templin C., Lüscher T.F. (2017). Cardiac magnetic resonance imaging in myocarditis reveals persistent disease activity despite normalization of cardiac enzymes and inflammatory parameters at 3-month follow-up. Circ. Heart Fail..

[191] Murtagh G., Laffin L.J., Beshai J.F., Maffessanti F., Bonham C.A., Patel A.V., Yu Z., Addetia K., Mor-Avi V., Moss J.D. (2016). Prognosis of myocardial damage in sarcoidosis patients with preserved left ventricular ejection fraction: Risk stratification using cardiovascular magnetic resonance. Circ. Cardiovasc. Imaging.

[192] Nensa F., Bamberg F., Rischpler C., Menezes L., Poeppel T.D., la Fougère C., Beitzke D., Rasul S., Loewe C., Nikolaou K. (2018). European Society of Cardiovascular Radiology (ESCR); European Association of Nuclear Medicine (EANM) Cardiovascular Committee. Hybrid cardiac imaging using PET/MRI: A joint position statement by the European Society of Cardiovascular Radiology (ESCR) and the European Association of Nuclear Medicine (EANM). Eur. Radiol..

[193] Lapinskas T., Pedrizzetti G., Stoiber L., Düngen H.D., Edelmann F., Pieske B., Kelle S. (2019). The Intraventricular hemodynamic forces estimated using routine CMR cine images: A new marker of the failing heart. JACC Cardiovasc. Imaging.

[194] Frey N., Meder B., Katus H.A. (2018). Left ventricular biopsy in the diagnosis of myocardial diseases. Circulation.

[195] Nakayama T., Murai S., Ohte N. (2018). Dilated cardiomyopathy with eosinophilic granulomatosis with polyangiitis in which active myocardial inflammation was only detected by endomyocardial biopsy. Intern. Med..

[196] Raafs A.G., Verdonschot J.A.J., Henkens M.T.H.M., Adriaans B.P., Wang P., Derks K., Abdul Hamid M.A., Knackstedt C., van Empel V.P.M., Díez J. (2021). The combination of carboxy-terminal propeptide of procollagen type I blood levels and late gadolinium enhancement at cardiac magnetic resonance provides additional prognostic information in idiopathic dilated cardiomyopathy—A multilevel assessment of myocardial fibrosis in dilated cardiomyopathy. Eur. J. Heart Fail..

[197] Leone O., Veinot J.P., Angelini A., Baandrup U.T., Basso C., Berry G., Bruneval P., Burke M., Butany J., Calabrese F. (2012). 2011 consensus statement on endomyocardial biopsy from the Association for European Cardiovascular Pathology and the Society for Cardiovascular Pathology. Cardiovasc. Pathol..

[198] Katzmann J.L., Schlattmann P., Rigopoulos A.G., Noutsias E., Bigalke B., Pauschinger M., Tschope C., Sedding D., Schulze P.C., Noutsias M. (2020). Meta-analysis on the immunohistological detection of inflammatory cardiomyopathy in endomyocardial biopsies. Heart Fail. Rev..

[199] Baughman K.L. (2006). Diagnosis of myocarditis: Death of Dallas criteria. Circulation.

[200] Andreoletti L., Leveque N., Boulagnon C., Brasselet C., Fornes P. (2009). Viral causes of human myocarditis. Arch. Cardiovasc. Dis..

[201] Badorff C., Knowlton K.U. (2004). Dystrophin disruption in enterovirus-induced myocarditis and dilated cardiomyopathy: From bench to bedside. Med. Microbiol. Immunol..

[202] Spieker M., Haberkorn S., Gastl M., Behm P., Katsianos S., Horn P., Jacoby C., Schnackenburg B., Reinecke P., Kelm M. (2017). Abnormal T2 mapping cardiovascular magnetic resonance correlates with adverse clinical outcome in patients with suspected acute myocarditis. J. Cardiovasc. Magn. Reson..

[203] Unterberg-Buchwald C., Ritter C.O., Reupke V., Wilke R.N., Stadelmann C., Steinmetz M., Schuster A., Hasenfuß G., Lotz J., Uecker M. (2017). Targeted endomyocardial biopsy guided by real-time cardiovascular magnetic resonance. J. Cardiovasc. Magn. Reson..

[204] Casella M., Pizzamiglio F., Dello Russo A., Carbucicchio C., Al-Mohani G., Russo E., Notarstefano P., Pieroni M., D’Amati G., Sommariva E. (2015). Feasibility of combined unipolar and bipolar voltage maps to improve sensitivity of endomyocardial biopsy. Circ. Arrhythm. Electrophysiol..

[205] Liang J.J., Hebl V.B., DeSimone C.V., Madhavan M., Nanda S., Kapa S., Maleszewski J.J., Edwards W.D., Reeder G., Cooper L.T. (2014). Electrogram guidance: A method to increase the precision and diagnostic yield of endomyocardial biopsy for suspected cardiac sarcoidosis and myocarditis. JACC Heart Fail..

[206] Konecny T., Noseworthy P.A., Kapa S., Cooper L.T., Mulpuru S.K., Sandhu G.S., Asirvatham S. (2015). Endomyocardial biopsy-integrating electrode at the bioptome tip. Ther. Adv. Cardiovasc. Dis..

[207] Vaidya V.R., Abudan A.A., Vasudevan K., Shantha G., Cooper L.T., Kapa S., Noseworthy P.A., Cha Y.-M., Asirvatham S.J., Deshmukh A.J. (2018). The efficacy and safety of electroanatomic mapping-guided endomyocardial biopsy: A systematic review. J. Interv. Card. Electrophysiol..

[208] Omote K., Naya M., Koyanagawa K., Aikawa T., Manabe O., Nagai T., Kamiya K., Kato Y., Komoriyama H., Kuzume M. (2019). ^18^F-FDG uptake of the right ventricle is an important predictor of histopathologic diagnosis by endomyocardial biopsy in patients with cardiac sarcoidosis. J. Nucl. Cardiol..

[209] Van Linthout S., Tschope C. (2018). Viral myocarditis: A prime example for endomyocardial biopsy-guided diagnosis and therapy. Curr. Opin. Cardiol..

[210] Lassner D., Kühl U., Siegismund C.S., Rohde M., Elezkurtaj S., Escher F., Tschöpe C., Gross U.M., Poller W., Schultheiss H.-P. (2014). Improved diagnosis of idiopathic giant cell myocarditis and cardiac sarcoidosis by myocardial gene expression profiling. Eur. Heart J..

[211] Hammer E., Darm K., Volker U. (2013). Characterization of the human myocardial proteome in dilated cardiomyopathy by label-free quantitative shotgun proteomics of heart biopsies. Methods Mol. Biol..

[212] Van Linthout S., Tschope C. (2017). Lost in markers? Time for phenomics and phenomapping in dilated cardiomyopathy. Eur. J. Heart Fail..

[213] Soler-Botija C., Galvez-Monton C., Bayes-Genis A. (2019). Epigenetic biomarkers in cardiovascular diseases. Front. Genet..

[214] Halliday B.P., Cleland JG F., Goldberger J.J., Prasad S.K. (2017). Personalizing risk stratification for sudden death in dilated cardiomyopathy: The past, present, and future. Circulation.

[215] Takeuchi S., Kawada J.I., Okuno Y., Horiba K., Suzuki T., Torii Y., Yasuda K., Numaguchi A., Kato T., Takahashi Y. (2018). Identification of potential pathogenic viruses in patients with acute myocarditis using next generation sequencing. J. Med. Virol..

[216] Reichl K., Kreykes S.E., Martin C.M., Shenoy C. (2018). Desmoplakin variant-associated arrhythmogenic cardiomyopathy presenting as acute myocarditis. Circ. Genom. Precis. Med..

[217] Calabrese F., Basso C., Carturan E., Valente M., Thiene G. (2006). Arrhythmogenic right ventricular cardiomyopathy/dysplasia: Is there a role for viruses?. Cardiovasc. Pathol..

[218] Lopez-Ayala J.M., Pastor-Quirante F., Gonzalez-Carrillo J., Lopez-Cuenca D., Sanchez-Munoz J.J., Oliva-Sandoval M.J., Gimeno J.R. (2015). Genetics of myocarditis in arrhythmogenic right ventricular dysplasia. Heart Rhythm.

[219] Protonotarios A., Wicks E., Ashworth M., Stephenson E., Guttmann O., Savvatis K., Sekhri N., Mohiddin S.A., Syrris P., Menezes L. (2019). Prevalence of ^18^Ffluorodeoxyglucose positron emission tomography abnormalities in patients with arrhythmogenic right ventricular cardiomyopathy. Int. J. Cardiol..

[220] Hata Y., Hirono K., Yamaguchi Y., Ichida F., Oku Y., Nishida N. (2019). Minimal inflammatory foci of unknown etiology may be a tentative sign of early stage inherited cardiomyopathy. Mod. Pathol..

[221] Belkaya S., Kontorovich A.R., Byun M., Mulero-Navarro S., Bajolle F., Cobat A., Josowitz R., Itan Y., Quint R., Lorenzo L. (2017). Autosomal recessive cardiomyopathy presenting as acute myocarditis. J. Am. Coll. Cardiol..

[222] Nappi F., Iervolino A., Avtaar Singh S.S., Chello M. (2021). MicroRNAs in Valvular Heart Diseases: Biological Regulators, Prognostic Markers and Therapeutical Targets. Int. J. Mol. Sci..

[223] Nappi F. (2024). Non-Coding RNA-Targeted Therapy: A State-of-the-Art Review. Int. J. Mol. Sci..

[224] Nappi F., Avtaar Singh S.S., Jitendra V., Alzamil A., Schoell T. (2023). The Roles of microRNAs in the Cardiovascular System. Int. J. Mol. Sci..

[225] Nenna A., Loreni F., Giacinto O., Chello C., Nappi P., Chello M., Nappi F. (2022). miRNAs in Cardiac Myxoma: New Pathologic Findings for Potential Therapeutic Opportunities. Int. J. Mol. Sci..

[226] Nappi F., Alzamil A., Avtaar Singh S.S., Spadaccio C., Bonnet N. (2023). Current Knowledge on the Interaction of Human Cytomegalovirus Infection, Encoded miRNAs, and Acute Aortic Syndrome. Viruses.

[227] Loreni F., Nenna A., Nappi F., Ferrisi C., Chello C., Lusini M., Vincenzi B., Tonini G., Chello M. (2024). miRNAs in the diagnosis and therapy of cardiac and mediastinal tumors: A new dawn for cardio-oncology?. Future Cardiol..

[228] Small E.M., Olson E.N. (2011). Pervasive roles of microRNAs in cardiovascular biology. Nature.

[229] Corsten M.F., Papageorgiou A., Verhesen W., Carai P., Lindow M., Obad S., Summer G., Coort S.L., Hazebroek M., van Leeuwen R. (2012). MicroRNA profiling identifies microRNA-155 as an adverse mediator of cardiac injury and dysfunction during acute viral myocarditis. Circ. Res..

[230] Kuehl U., Lassner D., Gast M., Stroux A., Rohde M., Siegismund C., Wang X., Escher F., Gross M., Skurk C. (2015). Differential cardiac microRNA expression predicts the clinical course in human enterovirus cardiomyopathy. Circ. Heart Fail..

[231] Navarro I.C., Ferreira F.M., Nakaya H.I., Baron M.A., Vilar-Pereira G., Pereira I.R., Silva A.M., Real J.M., De Brito T., Chevillard C. (2015). MicroRNA transcriptome profiling in heart of Trypanosoma cruzi-infected mice: Parasitological and cardiological outcomes. PLoS Negl. Trop. Dis..

[232] Corsten M.F., Dennert R., Jochems S., Kuznetsova T., Devaux Y., Hofstra L., Wagner D.R., Staessen J.A., Heymans S., Schroen B. (2010). Circulating microRNA-208b and microRNA-499 reflect myocardial damage in cardiovascular disease. Circ. Cardiovasc. Genet..

[233] Goldberg L., Tirosh-Wagner T., Vardi A., Abbas H., Pillar N., Shomron N., Nevo-Caspi Y., Paret G. (2018). Circulating microRNAs: A potential biomarker for cardiac damage, inflammatory response, and left ventricular function recovery in pediatric viral myocarditis. J. Cardiovasc. Transl. Res..

[234] Devaux Y., Vausort M., Goretti E., Nazarov P.V., Azuaje F., Gilson G., Corsten M.F., Schroen B., Lair M.L., Heymans S. (2012). Use of circulating microRNAs to diagnose acute myocardial infarction. Clin. Chem..

[235] Heidecker B., Kittleson M.M., Kasper E.K., Wittstein I.S., Champion H.C., Russell S.D., Hruban R.H., Rodriguez E.R., Baughman K.L., Hare J.M. (2011). Transcriptomic biomarkers for the accurate diagnosis of myocarditis. Circulation.

[236] Nie X., Li H., Wang J., Cai Y., Fan J., Dai B., Chen C., Wang D.W. (2021). Expression Profiles and Potential Functions of Long Non-Coding RNAs in the Heart of Mice with Coxsackie B3 Virus-Induced Myocarditis. Front. Cell. Infect. Microbiol..

[237] Chen P., Baldeviano G.C., Ligons D.L., Talor M.V., Barin J.G., Rose N.R., Cihakova D. (2012). Susceptibility to autoimmune myocarditis is associated with intrinsic differences in CD4(+) T cells. Clin. Exp. Immunol..

[238] Li J., Wang L., Wang S., Zhu H., Ye P., Xie A., Shen B., Liu C., Guo C., Fu Q. (2010). The Treg/Th17 imbalance in patients with idiopathic dilated cardiomyopathy. Scand. J. Immunol..

[239] Benincasa G., Mansueto G., Napoli C. (2019). Fluid-based assays and precision medicine of cardiovascular diseases: The ‘hope’ for Pandora’s box?. J. Clin. Pathol..

[240] Kennel P.J., Saha A., Maldonado D.A., Givens R., Brunjes D.L., Castillero E., Zhang X., Ji R., Yahi A., George I. (2018). Serum exosomal protein profiling for the non-invasive detection of cardiac allograft rejection. J. Heart Lung Transpl..

[241] Ponikowski P., Voors A.A., Anker S.D., Bueno H., Cleland J.G., Coats A.J., Falk V., González-Juanatey J.R., Harjola V.P., Jankowska E.A. (2016). 2016 ESC Guidelines for the diagnosis and treatment of acute and chronic heart failure: The Task Force for the diagnosis and treatment of acute and chronic heart failure of the European Society of Cardiology (ESC). Developed with the special contribution of the Heart Failure Association (HFA) of the ESC. Eur. J. Heart Fail..

[242] Peretto G., Sala S., Rizzo S., Palmisano A., Esposito A., De Cobelli F., Campochiaro C., De Luca G., Foppoli L., Dagna L. (2020). Ventricular arrhythmias in myocarditis: Characterization and relationships with myocardial inflammation. J. Am. Coll. Cardiol..

[243] Baksi A.J., Kanaganayagam G.S., Prasad S.K. (2015). Arrhythmias in viral myocarditis and pericarditis. Card. Electrophysiol. Clin..

[244] Cooper L.T., Berry G.J., Shabetai R. (1997). Idiopathic giant-cell myocarditis—Natural history and treatment. N. Engl. J. Med..

[245] Birnie D.H., Sauer W.H., Bogun F., Cooper J.M., Culver D.A., Duvernoy C.S., Judson M.A., Kron J., Mehta D., Cosedis Nielsen J. (2014). HRS expert consensus statement on the diagnosis and management of arrhythmias associated with cardiac sarcoidosis. Heart Rhythm.

[246] Imazio M., Trinchero R. (2008). Myopericarditis: Etiology, management, and prognosis. Myopericarditis: Etiology, management, and prognosis. Int. J. Cardiol..

[247] Adegbala O., Olagoke O., Akintoye E., Adejumo A.C., Oluwole A., Jara C., Williams K., Briasoulis A., Afonso L. (2019). Predictors, burden, and the impact of arrhythmia on patients admitted for acute myocarditis. Am. J. Cardiol..

[248] Maron B.J., Udelson J.E., Bonow R.O., Nishimura R.A., Ackerman M.J., Estes N.A., Cooper L.T., Link M.S., Maron M.S. (2015). Eligibility and disqualification recommendations for competitive athletes with cardiovascular abnormalities: Task Force 3: Hypertrophic cardiomyopathy, arrhythmogenic right ventricular cardiomyopathy and other cardiomyopathies, and myocarditis: A scientific statement from the American Heart Association and American College of Cardiology. Circulation.

[249] Zorzi A., Perazzolo Marra M., Rigato I., De Lazzari M., Susana A., Niero A., Pilichou K., Migliore F., Rizzo S., Giorgi B. (2016). Nonischemic left ventricular scar as a substrate of life-threatening ventricular arrhythmias and sudden cardiac death in competitive athletes. Circ. Arrhythm. Electrophysiol..

[250] Steinke K. (2013). Coxsackievirus B3 modulates cardiac ion channels Coxsackievirus B3 modulates cardiac ion channels. FASEB J..

[251] Priori S.G., Blomström-Lundqvist C., Mazzanti A., Blom N., Borggrefe M., Camm J., Elliott P.M., Fitzsimons D., Hatala R., Hindricks G. (2015). 2015 ESC Guidelines for the management of patients with ventricular arrhythmias and the prevention of sudden cardiac death: The Task Force for the Management of Patients with Ventricular Arrhythmias and the Prevention of Sudden Cardiac Death of the European Society of Cardiology (ESC). Endorsed by: Association for European Paediatric and Congenital Cardiology (AEPC). Europace.

[252] Sheppard R., Mather P.J., Alexis J.D., Starling R.C., Boehmer J.P., Thohan V., Pauly D.F., Markham D.W., Zucker M., Kip K.E. (2012). Implantable cardiac defibrillators and sudden death in recent onset nonischemiccardiomyopathy: Results from IMAC2. J. Card. Fail..

[253] Chung M.K. (2014). The role of the wearable cardioverter defibrillator in clinical practice. Cardiol. Clin..

[254] Halle M., Binzenhöfer L., Mahrholdt H., Johannes Schindler M., Esefeld K., Tschöpe C. (2021). Myocarditis in athletes: A clinical perspective. Eur. J. Prev. Cardiol..

[255] Wojnicz R., Nowalany-Kozielska E., Wojciechowska C., Glanowska G., Wilczewski P., Niklewski T., Zembala M., Polonski L., Rozek M.M., Wodniecki J. (2001). Randomized, placebo-controlled study for immunosuppressive treatment of inflammatory dilated cardiomyopathy: Two-year follow-up results. Circulation.

[256] Merken J., Hazebroek M., Van Paassen P., Verdonschot J., Van Empel V., Knackstedt C., Abdul Hamid M., Seiler M., Kolb J., Hoermann P. (2018). Immunosuppressive therapy improves both short-and long-term prognosis in patients with virus-negative nonfulminant inflammatory cardiomyopathy. Circ. Heart Fail..

[257] Kleinert S., Weintraub R.G., Wilkinson J.L., Chow C.W. (1997). Myocarditis in children with dilated cardiomyopathy: Incidence and outcome after dual therapy immunosuppression. J. Heart Lung Transpl..

[258] De Luca G., Campochiaro C., Sartorelli S., Peretto G., Sala S., Palmisano A., Esposito A., Candela C., Basso C., Rizzo S. (2020). Efficacy and safety of mycophenolate mofetil in patients with virus-negative lymphocytic myocarditis: A prospective cohort study. J. Autoimmun..

[259] Felix S.B., Staudt A., Dörffel W.V., Stangl V., Merkel K., Pohl M., Döcke W.D., Morgera S., Neumayer H.H., Wernecke K.D. (2000). Hemodynamic effects of immunoadsorption and subsequent immunoglobulin substitution in dilated cardiomyopathy: Three-month results from a randomized study. J. Am. Coll. Cardiol..

[260] Trimpert C., Herda L.R., Eckerle L.G., Pohle S., Müller C., Landsberger M., Felix S.B., Staudt A. (2010). Immunoadsorption in dilated cardiomyopathy: Long-term reduction of cardiodepressant antibodies. Eur. J. Clin. Invest..

[261] Dandel M., Wallukat G., Englert A., Lehmkuhl H.B., Knosalla C., Hetzer R. (2012). Long-term benefits of immunoadsorption in β(1)-adrenoceptor autoantibody-positive transplant candidates with dilated cardiomyopathy. Eur. J. Heart Fail..

[262] Kronbichler A., Brezina B., Quintana L.F., Jayne D.R. (2016). Efficacy of plasma exchange and immunoadsorption in systemic lupus erythematosus and antiphospholipid syndrome: A systematic review. Autoimmun. Rev..

[263] Yamaji K. (2017). Immunoadsorption for collagen and rheumatic diseases. Transfus. Apher. Sci..

[264] Staudt A., Schäper F., Stangl V., Plagemann A., Böhm M., Merkel K., Wallukat G., Wernecke K.D., Stangl K., Baumann G. (2001). Immunohistological changes in dilated cardiomyopathy induced by immunoadsorption therapy and subsequent immunoglobulin substitution. Circulation.

[265] US National Library of Medicine (2018). ClinicalTrials.gov. https://clinicaltrials.gov/ct2/show/NCT00558584.

[266] Dungen H.D., Dordevic A., Felix S.B., Pieske B., Voors A.A., McMurray J.J.V., Butler J. (2020). β1-Adrenoreceptor autoantibodies in heart failure: Physiology and therapeutic implications. Circ. Heart Fail..

[267] Schultheiss H.P., Piper C., Sowade O., Waagstein F., Kapp J.F., Wegscheider K., Groetzbach G., Pauschinger M., Escher F., Arbustini E. (2016). Betaferon in chronic viral cardiomyopathy (BICC) trial: Effects of interferon-β treatment in patients with chronic viral cardiomyopathy. Clin. Res. Cardiol..

[268] Kuhl U., Lassner D., Wallaschek N., Gross U.M., Krueger G.R., Seeberg B., Kaufer B.B., Escher F., Poller W., Schultheiss H.P. (2015). Chromosomally integrated human herpesvirus 6 in heart failure: Prevalence and treatment. Eur. J. Heart Fail..

[269] Tschope C., Elsanhoury A., Schlieker S., Van Linthout S., Kuhl U. (2019). Immunosuppression in inflammatory cardiomyopathy and parvovirus B19 persistence. Eur. J. Heart Fail..

[270] Ameling S., Bhardwaj G., Hammer E., Beug D., Steil L., Reinke Y., Weitmann K., Grube M., Trimpert C., Klingel K. (2016). Changes of myocardial gene expression and protein composition in patients with dilated cardiomyopathy after immunoadsorption with subsequent immunoglobulin substitution. Basic Res. Cardiol..

[271] McNamara D.M., Holubkov R., Starling R.C., Dec G.W., Loh E., Torre-Amione G., Gass A., Janosko K., Tokarczyk T., Kessler P. (2001). Controlled trial of intravenous immune globulin in recent-onset dilated cardiomyopathy. Circulation.

[272] Maisch B., Hufnagel G., Kölsch S., Funck R., Richter A., Rupp H., Herzum M., Pankuweit S. (2004). Treatment of inflammatory dilated cardiomyopathy and (peri)myocarditis with immunosuppression and i.v. immunoglobulins. Herz.

[273] Sudano I., Spieker L.E., Noll G., Corti R., Weber R., Lüscher T.F. (2006). Cardiovascular disease in HIV infection. Am. Heart J..

[274] Baik S.H., Jeong H.S., Kim S.J., Yoon Y.K., Sohn J.W., Kim M.J. (2015). A case of influenza associated fulminant myocarditis successfully treated with intravenous peramivir. Infect. Chemother..

[275] Ito N., Sato M., Momoi N., Aoyagi Y., Endo K., Chishiki M., Kawasaki Y., Hosoya M. (2015). Influenza A H1N1 pdm09-associated myocarditis during zanamivir therapy. Pediatr. Int..

[276] Sanders J.M., Monogue M.L., Jodlowski T.Z., Cutrell J.B. (2020). Pharmacologic treatments for coronavirus disease 2019 (COVID-19): A review. JAMA.

[277] Mann D.L. (2004). Targeted anticytokine therapy in patients with chronic heart failure: Results of the Randomized Etanercept Worldwide Evaluation (RENEWAL). Circulation.

[278] Tschöpe C., Van Linthout S., Jäger S., Arndt R., Trippel T., Müller I., Elsanhoury A., Rutschow S., Anker S.D., Schultheiss H.P. (2020). Modulation of the acute defence reaction by eplerenone prevents cardiac disease progression in viral myocarditis. ESC Heart Fail..

[279] Lee W.S., Erdelyi K., Matyas C., Mukhopadhyay P., Varga Z.V., Liaudet L., Haskú G., Čiháková D., Mechoulam R., Pacher P. (2016). Cannabidiol limits T cell-mediated chronic autoimmune myocarditis: Implications to autoimmune disorders and organ transplantation. Mol. Med..

[280] Branchereau M., Burcelin R., Heymes C. (2019). The gut microbiome and heart failure: A better gut for a better heart. Rev. Endocr. Metab. Disord..

[281] Pinkert S., Westermann D., Wang X., Klingel K., Dörner A., Savvatis K., Grössl T., Krohn S., Tschöpe C., Zeichhardt H. (2009). Prevention of cardiac dysfunction in acute coxsackievirus B3 cardiomyopathy by inducible expression of a soluble coxsackievirus-adenovirus receptor. Circulation.

[282] Pinkert S., Dieringer B., Klopfleisch R., Savvatis K., Van Linthout S., Pryshliak M., Tschöpe C., Klingel K., Kurreck J., Beling A. (2019). Early treatment of coxsackievirus B3-infected animals with soluble coxsackievirusadenovirus receptor inhibits development of chronic coxsackievirus B3 cardiomyopathy. Circ. Heart Fail..

[283] Kraft Erdenesukh T., Sauter M., Tschope C., Klingel K. (2019). Blocking the IL-1β signalling pathway prevents chronic viral myocarditis and cardiac remodeling. Basic Res. Cardiol..

[284] Brucato A., Imazio M., Gattorno M., Lazaros G., Maestroni S., Carraro M., Finetti M., Cumetti D., Carobbio A., Ruperto N. (2016). Effect of anakinra on recurrent pericarditis among patients with colchicine resistance and corticosteroid dependence: The AIRTRIP randomized clinical trial. JAMA.

[285] Scott I.C., Hajela V., Hawkins P.N., Lachmann H.J. (2011). A case series and systematic literature review of anakinra and immunosuppression in idiopathic recurrent pericarditis. J. Cardiol. Cases.

[286] Rodriguez-Gonzalez M., Ruiz-Gonzalez E., Castellano-Martinez A. (2019). Anakinra as rescue therapy for steroid-dependent idiopathic recurrent pericarditis in children: Case report and literature review. Cardiol. Young.

[287] US National Library of Medicine (2024). ClinicalTrials.gov. https://clinicaltrials.gov/ct2/show/NCT03018834.

[288] US National Library of Medicine (2023). ClinicalTrials.gov. https://clinicaltrials.gov/ct2/show/NCT03737110.

[289] Li Z., Yue Y., Xiong S. (2013). Distinct Th17 inductions contribute to the gender bias in CVB3-induced myocarditis. Cardiovasc. Pathol..

[290] Abou-El-Enein M., Volk H.D., Reinke P. (2017). Clinical development of cell therapies: Setting the stage for academic success. Clin. Pharmacol. Ther..

[291] Koch M., Savvatis K., Scheeler M., Dhayat S., Bonaventura K., Pohl T., Riad A., Bulfone-Paus S., Schultheiss H.P., Tschöpe C. (2010). Immunosuppression with an interleukin-2 fusion protein leads to improved LV function in experimental ischemic cardiomyopathy. Int. Immunopharmacol..

[292] Fan M.Y., Low J.S., Tanimine N., Finn K.K., Priyadharshini B., Germana S.K., Kaech S.M., Turka L.A. (2018). Differential roles of IL-2 signaling in developing versus mature Tregs. Cell Rep..

[293] Van Linthout S., Savvatis K., Miteva K., Peng J., Ringe J., Warstat K., Schmidt-Lucke C., Sittinger M., Schultheiss H.P., Tschöpe C. (2011). Mesenchymal stem cells improve murine acute coxsackievirus B3-induced myocarditis. Eur. Heart J..

[294] Hare J.M., DiFede D.L., Rieger A.C., Florea V., Landin A.M., El-Khorazaty J., Khan A., Mushtaq M., Lowery M.H., Byrnes J.J. (2017). Randomized comparison of allogeneic versus autologous mesenchymal stem cells for nonischemic dilated cardiomyopathy: POSEIDONDCM trial. J. Am. Coll. Cardiol..

[295] Rieger A.C., Myerburg R.J., Florea V., Tompkins B.A., Natsumeda M., Premer C., Khan A., Schulman I.H., Vidro-Casiano M., DiFede D.L. (2019). Genetic determinants of responsiveness to mesenchymal stem cell injections in non-ischemic dilated cardiomyopathy. EBioMedicine.

[296] Lorusso R., Centofanti P., Gelsomino S., Barili F., Di Mauro M., Orlando P., Botta L., Milazzo F., Actis Dato G., Casabona R. (2016). Venoarterial extracorporeal membrane oxygenation for acute fulminant myocarditis in adult patients: A 5-year multi-institutional experience. Ann. Thorac. Surg..

[297] Kapur N.K., Davila C.D., Jumean M.F. (2017). Integrating interventional cardiology and heart failure management for cardiogenic shock. Interv. Cardiol. Clin..

[298] Li S., Xu S., Li C., Ran X., Cui G., He M., Miao K., Zhao C., Yan J., Hui R. (2019). A life support-based comprehensive treatment regimen dramatically lowers the in-hospital mortality of patients with fulminant myocarditis: A multiple center study. Sci. China Life Sci..

[299] Kapur N.K., Esposito M.L., Bader Y., Morine K.J., Kiernan M.S., Pham D.T., Burkhoff D. (2017). Mechanical circulatory support devices for acute right ventricular failure. Circulation.

[300] Annamalai S.K., Esposito M.L., Jorde L., Schreiber T., AHall S., O’Neill W.W., Kapur N.K. (2018). The Impella microaxial flow catheter is safe and effective for treatment of myocarditis complicated by cardiogenic shock: An analysis from the global cVAD registry. J. Card. Fail..

[301] Spillmann F. (2019). Mode-of-action of the PROPELLA concept in fulminant myocarditis. Mode-of-action of the PROPELLA concept in fulminant myocarditis. Eur. Heart J..

[302] Sun M., Ishii R., Okumura K., Krauszman A., Breitling S., Gomez O., Hinek A., Boo S., Hinz B., Connelly K.A. (2018). Experimental right ventricular hypertension induces regional β1-integrin-mediated transduction of hypertrophic and profibrotic right and left ventricular signaling. J. Am. Heart Assoc..

[303] Lindner D. (2014). Cardiac fibroblasts support cardiac inflammation in heart failure. Basic Res. Cardiol..

[304] Levin H.R., Oz M.C., Chen J.M., Packer M., Rose E.A., Burkhoff D. (1995). Reversal of chronic ventricular dilation in patients with end-stage cardiomyopathy by prolonged mechanical unloading. Circulation.

[305] Hata J.A., Williams M.L., Schroder J.N., Lima B., Keys J.R., Blaxall B.C., Petrofski J.A., Jakoi A., Milano C.A., Koch W.J. (2006). Lymphocyte levels of GRK2 (βARK1) mirror changes in the LVAD-supported failing human heart: Lower GRK2 associated with improved β-adrenergic signaling after mechanical unloading. J. Card. Fail..

[306] Tschope C., Van Linthout S., Klein O., Mairinger T., Krackhardt F., Potapov E.V., Schmidt G., Burkhoff D., Pieske B., Spillmann F. (2019). Mechanical unloading by fulminant myocarditis: LV-IMPELLA, ECMELLA, BI-PELLA, and PROPELLA concepts. J Cardiovasc. Transl. Res..

[307] Chaparro S.V., Badheka A., Marzouka G.R., Tanawuttiwat T., Ahmed F., Sacher V., Pham S.M. (2012). Combined use of Impella left ventricular assist device and extracorporeal membrane oxygenation as a bridge to recovery in fulminant myocarditis. ASAIO J..

[308] Pappalardo F., Schulte C., Pieri M., Schrage B., Contri R., Soeffker G., Greco T., Lembo R., Müllerleile K., Colombo A. (2017). Concomitant implantation of Impella((R)) on top of veno-arterial extracorporeal membrane oxygenation may improve survival of patients with cardiogenic shock. Eur. J. Heart Fail..

[309] Pappalardo F., Scandroglio A.M., Latib A. (2018). Full percutaneous biventricular support with two Impella pumps: The Bi-Pella approach. ESC Heart Fail..

[310] Tominaga Y., Toda K., Miyagawa S., Yoshioka D., Kainuma S., Kawamura T., Kawamura A., Nakamoto K., Sakata Y., Sawa Y. (2021). Total percutaneous biventricular assist device implantation for fulminant myocarditis. J. Artif. Organs.

[311] Fiedler A. (1900). Uber akute interstitielle Myokarditis. Zentralblatt Inn. Med..

[312] Sakakibara S., Konno S. (1962). Endomyocardial biopsy. Jpn. Heart J..

[313] Laser J.A., Fowles R.E., Mason J.W. (1985). Endomyocardial biopsy. Cardiovasc. Clin..

[314] Mason J.W., O’Connell J.B. (1989). Clinical merit of endomyocardial biopsy. Circulation.

[315] Zanatta A., Carturan E., Rizzo S., Basso C., Thiene G. (2019). Story telling of myocarditis. Int. J. Cardiol..

[316] Thiene G. (2024). Storytelling of Myocarditis. Biomedicines.

