# Supplementary material for: Myocarditis and Inflammatory Cardiomyopathy in Dilated Heart Failure
Source: Viruses. 2025 Mar 27;17(4):484. doi: 10.3390/v17040484 (PMC12031395; doi:10.3390/v17040484)
Supplement: Supplementary file 1 [file viruses-17-00484-s001.zip › viruses-3482853-supplementary.pdf]

Supplementary Material

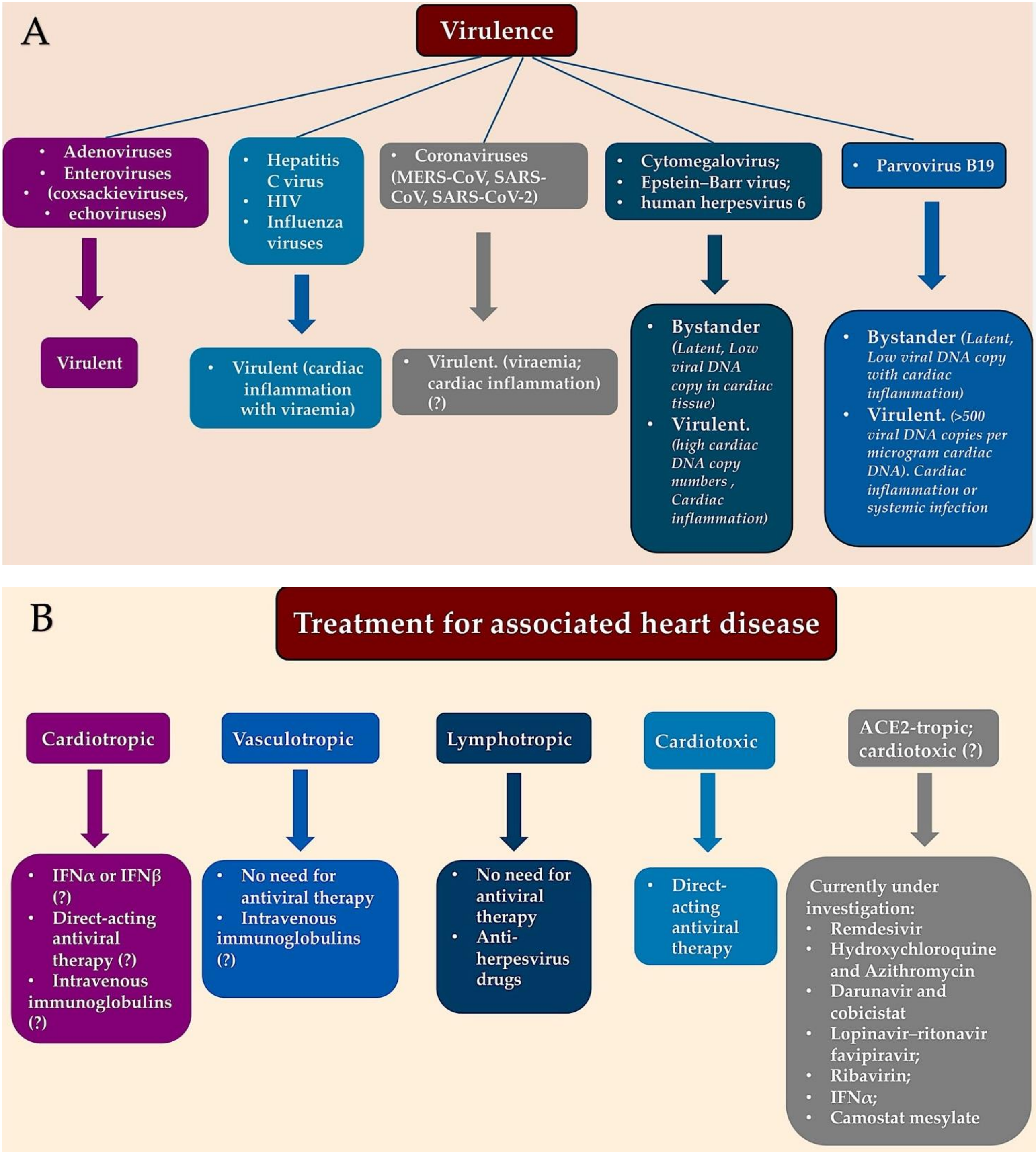

Figure S1. The following illustration is of viruses associated with myocarditis and inflammatory cardiomyopathy **(A,B). (A):** The virulence may be attributed to the specific type of viral infection. The occurrence of viremia or latency is a crucial consideration in determining the virulence of a viral infection. (**B):** correlation between the application of medication and the sustained infection caused by a specific viral agent. Abbreviations; '(?)' indicates unclear, needs further investigation; ACE2, angiotensin-converting enzyme 2; dsDNA, double- stranded DNA; HIV, human immunodeficiency virus; MERS-CoV, Middle East respiratory syndrome coronavirus; SARS-CoV, severe acute respiratory syndrome coronavirus; ssDNA, single- stranded DNA; (+)ssRNA, positive-sense single- stranded RNA [4,10,21-35].

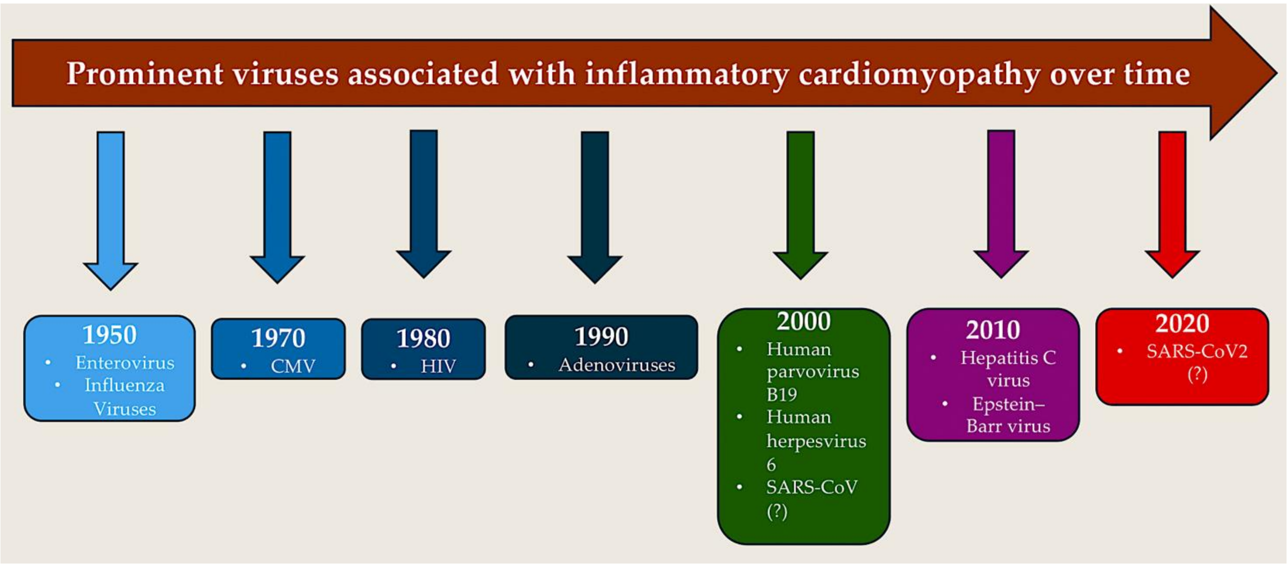

Figure S2. As time progresses, there has been an upward trend in the number of identified viruses linked with inflammatory cardiomyopathy. This evolution is influenced by two factors: firstly, the intentional detection of a broader repertoire of viruses over time and, secondly, the occurrence of novel viruses or virus genotypes in the heart. However, further investigation is required to elucidate the association between severe acute respiratory syndrome coronavirus (SARS-CoV) and SARS-CoV-2 with inflammatory cardiomyopathy. The utilisation of the symbol '(?)' signifies uncertainty and underscores the necessity for further investigation. [26]

**Table S1. Future priorities and research gaps to explore the viral function**
• Viral detection methods must be improved because current methods are not sensitive enough for detecting viral genomes in human cardiac specimens**.**
• Use next-generation sequencing (NGS) and metagenomics methods to detect pathogens accurately. (64). It is vital to take this strategy because there is currently a lack of information about mutant viruses and 'new' viruses associated with inflammation of the heart muscle.
• Learn about how important viral load is in terms of causing disease and how it can be predicted.
• Find out how patients' genes and sex affect how viral myocarditis progresses and how it ends up.
• Know the difference between the following types of viral infection in the heart: active, persistent and/or latent.
• Create vaccines to protect people against viruses that cause heart problems.
• Create lists of information to see how common different types of viruses are in people with acute myocarditis (a heart muscle inflammation) and chronic inflammatory cardiomyopathy (a long-term heart muscle inflammation), and to see the difference between children and adults.
• Find out how SARS-CoV-2 causes heart disease.

**Table S2.** Future priorities and research gaps to explore the function of immune cells
• Find out more about how the immune cell's response changes from protecting the body to hurting it.
• Create information about the number of Treg cells compared to TH17 cells in patients with myocarditis or inflammatory cardiomyopathy.
• Learn why some individuals with autoimmune diseases or problems with their immune cells do not get heart inflammation or heart muscle problems.
• Find out why only some individuals with viral myopathy or inflammation of the heart muscle show autoimmunity or immune cell responses that are not normal.
• Learn how to target the immune system in a way that does not put the body at risk.
• Find out more about how neutrophils play a part in viral myocarditis.
• Find out more about the genetic and environmental factors that cause immune cells to respond in the wrong way.
• Look at whether genetic cardiomyopathies are connected to immune cells.
• Learn how research on immune cells in the heart from experiments on mice can be used to understand people.

Table S3. Technological Advancements, Clinical Applications and Cost Considerations.

- At the moment, the methods used to take pictures of the heart are not very good at predicting what will happen to patients with heart inflammation.
- At the moment, the imaging methods that are available cannot show us what is causing heart inflammation (for example, whether it is a virus). PET is the only one that can show us what is happening with sarcoid in the heart.
- Create a set of standard procedures for diagnosing heart problems using different types of cardiac MRI equipment.
- Create information about advanced MRI scans of the heart and how useful they are for predicting problems (including measurements of heart muscle strain, and T1 and T2 mapping, 4D flow and LV kinetic energy, and blood pressure). Do this for patients with myocarditis.
- Check for microvascular disease using a special type of heart scan (stress cardiac MRI) in patients who are showing signs of acute myocardial inflammation.
- Do studies to see how useful it is to combine different imaging methods for diagnosing and treating patients with acute or chronic myocarditis.
- Assess the cost-benefit, cost-effectiveness and budget impact models, using the right imaging methods for dealing with acute or chronic heart inflammation.
- Create a score for heart inflammation using MRI scans.

Table S4. Future priorities and research gaps for treating patients

▪ **Management of HF and arrhythmias**

- Look at how important traditional heart medication is for preventing heart failure in patients with myocarditis.
- Explain how LVEF can be used to predict the risk of sudden death in patients with myocarditis.
- Different types of heart inflammation can lead to sudden heart failure (e.g. bacterial versus immune-mediated).
- Decide the best time to put in the pacemaker.
- Look at how a wearable cardioverter-defibrillator works in patients with myocarditis who have a preserved LVEF and show signs of significant heart rhythm problems.
- Come up with a way to divide patients into groups based on the risk of sudden death in patients with myocarditis.
- Do trials to see how well antiarrhythmic drugs work for patients with heart inflammation.
- Find out how exercise affects the chance of sudden cardiac death and heart failure [254].

▪ **Drugs and biologics**

- Carry out large, forward-looking, randomised control studies to explore new or existing (repurposed) treatments that reduce or modify the immune system in patients with heart inflammation or heart muscle inflammation.
- Do trials at different hospitals that use EMG-guided or MRI to see how long patients should take conventional HF drugs, especially ones that are still being checked to make sure they can be used in patients.
- Carry out clinical studies that take into account the known differences between the sexes [33,34], in terms of immune responses and outcomes in patients with myocarditis [271].

▪ **New types of administered medications and MCS**

- Create new information from data collected in several hospitals to see if we can create simple rules to help us look after patients with a serious heart muscle disease.
- Find the best MCS or combination of MCS that allow the best blood flow and pressure relief in the left ventricle.
- Do random trials to see how well immunosuppressive treatments work for patients with a serious heart muscle disease who are on MCS.
